# Spectroscopic insights into multi-phase protein crystallization in complex lysate using Raman spectroscopy and a particle-free bypass

**DOI:** 10.3389/fbioe.2024.1397465

**Published:** 2024-05-15

**Authors:** Christina Henriette Wegner, Sebastian Mathis Eming, Brigitte Walla, Daniel Bischoff, Dirk Weuster-Botz, Jürgen Hubbuch

**Affiliations:** ^1^ Institute of Process Engineering in Life Sciences, Section IV: Biomolecular Separation Engineering, Karlsruhe Institute of Technology (KIT), Karlsruhe, Germany; ^2^ Institute of Biochemical Engineering, Technical University of Munich, Garching, Germany

**Keywords:** partial least squares regression (PLS), Raman spectroscopy, process analytical technology (PAT), protein crystallization, ultraviolet-visible light (UV/Vis) spectroscopy, principal component analyses (PCA), capture process, *E. coli* lysate

## Abstract

Protein crystallization as opposed to well-established chromatography processes has the benefits to reduce production costs while reaching a comparable high purity. However, monitoring crystallization processes remains a challenge as the produced crystals may interfere with analytical measurements. Especially for capturing proteins from complex feedstock containing various impurities, establishing reliable process analytical technology (PAT) to monitor protein crystallization processes can be complicated. In heterogeneous mixtures, important product characteristics can be found by multivariate analysis and chemometrics, thus contributing to the development of a thorough process understanding. In this project, an analytical set-up is established combining offline analytics, on-line ultraviolet visible light (UV/Vis) spectroscopy, and in-line Raman spectroscopy to monitor a stirred-batch crystallization process with multiple phases and species being present. As an example process, the enzyme *Lactobacillus kefir* alcohol dehydrogenase (L*k*ADH) was crystallized from clarified Escherichia coli (*E. coli*) lysate on a 300 mL scale in five distinct experiments, with the experimental conditions changing in terms of the initial lysate solution preparation method and precipitant concentration. Since UV/Vis spectroscopy is sensitive to particles, a cross-flow filtration (cross-flow filtration)-based bypass enabled the on-line analysis of the liquid phase providing information on the lysate composition regarding the nucleic acid to protein ratio. A principal component analysis (PCA) of *in situ* Raman spectra supported the identification of spectra and wavenumber ranges associated with productspecific information and revealed that the experiments followed a comparable, spectral trend when crystals were present. Based on preprocessed Raman spectra, a partial least squares (PLS) regression model was optimized to monitor the target molecule concentration in real-time. The off-line sample analysis provided information on the crystal number and crystal geometry by automated image analysis as well as the concentration of *Lk*ADH and host cell proteins (HCPs) In spite of a complex lysate suspension containing scattering crystals and various impurities, it was possible to monitor the target molecule concentration in a heterogeneous, multi-phase process using spectroscopic methods. With the presented analytical set-up of off-line, particle-sensitive on-line, and in-line analyzers, a crystallization capture process can be characterized better in terms of the geometry, yield, and purity of the crystals.

## 1 Introduction

Proteins, e.g., biopharmaceuticals, enzymes, and other biologically active molecules, offer a wide range of therapeutic applications and have reinvented the treatment of various diseases and disorders. Keys to the success of biological substances are efficient production, isolation, and purification using mostly chromatography as an expensive standard technique to ensure a high purity. Alternatively, other downstream processes, e.g., protein crystallization (Smejkal et al., 2013) or precipitation (Perosa et al., 1990; McDonald et al., 2009), can be developed. They are easier to scale, can achieve high purity and yield, and decrease production costs while maintaining high productivity. Whereas protein crystallization is traditionally associated with fundamental knowledge of the protein structure, the application for formulation and purification reasons has drawn more interests in the past years. Efforts focused on reducing the number of process steps to save both time and resources in the production of biological substances. For formulation, crystalline suspensions are beneficial due to their lower viscosity at high product concentration (Yang et al., 2003), higher stability (Shenoy et al., 2001), and potentially controlled release properties (Brader et al., 2002).

Saturation is the primary cause behind crystallization (Haas and Drenth, 1999) and it is influenced by a variety of environmental factors, e.g., protein concentration (Forsythe et al., 1997; Haas and Drenth, 1999), pH (Forsythe et al., 1997; Kantardjieff and Rupp, 2004), precipitant concentration (Chayen et al., 1988; Vivarès et al., 2002; Rakel et al., 2015), and temperature (Haas and Drenth, 1999; Martínez-Caballero et al., 2016). Compared to crystals of a chemical substance, the larger size of a biological molecule increases the complexity of the protein crystal. Therefore, extensive empirical screenings (Haas and Drenth, 1999; Asherie, 2004), precise, automated high-throughput (HT) techniques (Baumgartner et al., 2015; Huettmann et al., 2015), and HT analytics (Klijn and Hubbuch, 2020; Klijn and Hubbuch, 2021; Wegner et al., 2022; Wegner and Hubbuch, 2022) are essential to find optimal process conditions.

In the past, protein engineering made it possible to produce proteins with different capabilities or processing properties, e.g., increased crystallizability and solubility behavior (Lawson DM et al., 1991; McElroy et al., 1992; Charron et al., 2002). Increased crystallizability in particular may make protein crystallization attractive for larger production scales due to its higher productivity and higher probability to form crystals (Nowotny et al., 2019). Research on the micro-liter (McElroy et al., 1992; Charron et al., 2002; Nowotny et al., 2019) and milli-liter scales (Grob et al., 2020; Walla et al., 2021) has confirmed that protein crystal contacts in an enzyme can be improved to increase crystal occurrence and yield in pure protein solutions or clarified harvest, thus leading to a high product purity. In practice, harvest broth from biotechnological processes involves mixtures of proteins, impurities, and only a small quantity of the target molecule. While a lot of research was reported on scaled-up protein crystallization in solutions containing only traces of impurities or even none (Judge et al., 1995; Lee et al., 2000; Peters et al., 2005; Hebel et al., 2013; Yang et al., 2019; Chen et al., 2021; Tian et al., 2023; Jul-Jørgensen et al., 2024), the challenges imposed by complex solutions in capture processes have received relatively little attention (Takakura et al., 2006; Smejkal et al., 2013; Hekmat et al., 2017; Grob et al., 2020).

As suggested by the Food and Drug Administration (FDA) (Food and Drug Administration, 2004), process analytical technology (PAT) is crucial to ensuring the product safety for the patient and the quality of a pharmaceutical manufacturing process. To this end, the critical process parameters and quality attributes need to be controlled by selecting real-time analytics and suitable variables. Possible real-time process analyzers are spectroscopic measurements which are commonly applied in biotechnological processes. A lot of PAT research focuses on the particle-sensitive ultraviolet visible light (UV/Vis) spectroscopy (Brestrich et al., 2015; Andris et al., 2018), water-sensitive fourier-transform infrared spectroscopy (FTIR) (Cornel et al., 2008; Sanden et al., 2019) of process solutions with lower concentration (Hansen et al., 2011; Brestrich et al., 2015). These restrictions impose challenges when multiple phases and heterogeneous mixtures need to be monitored during a protein crystallization process in an aqueous environment. Especially when particles caused by aggregation, precipitation, or crystallization are present, PAT faces difficulties in choosing an adequate process analyzer. Light scattering, the heterogeneity of the suspension, and size distribution may affect the measurement and need to be considered for the data analysis. Raman spectroscopy may be a possible solution to this problem as it was shown to monitor crystallization processes of chemical target molecules (Hu et al., 2005; Simone et al., 2014a; Jul-Jørgensen et al., 2024) or the enzyme lysozyme (Mercado et al., 2008) out of pure component solutions (Févotte, 2007). When examining heterogeneous, more complex solutions, as e.g., in upstream processes (Berry et al., 2016; Esmonde-White et al., 2022), Raman spectroscopy has demonstrated its suitability for process monitoring despite possible interferences caused by sample turbidity (Esmonde-White et al., 2017), stirring, temperature (Wan et al., 2016), and pH fluctuation (Berry et al., 2016). In this context, the integration of Raman spectroscopy promises to improve our understanding of protein crystallization processes in heterogeneous mixtures. As an alternative, UV/Vis spectroscopy is a promising analytical tool when implemented in-line with an attenuated total reflectance (ATR) probe in pure solution crystallization processes (Bakar et al., 2009; Saleemi et al., 2012a; Simone et al., 2014b; Tacsi et al., 2020; Tian et al., 2023) and is often used for strongly absorbing solutions. UV/Vis transmission measurements with variable path length (VP) technology are more flexible in terms of solution absorption and was used when molecule concentration varied during the process (Brestich et al., 2018; Rolinger et al., 2020; Bhangale et al., 2022) similar to crystallization processes. However, this technique is prone to particle scattering and solid crystalline particles would interfere with the measurements.

Due to high correlation within the data set, the spectra produced by the above stated techniques are commonly processed using chemometric techniques, e.g., principal component analysis (PCA) (Simone et al., 2014a), partial least squares (PLS) (Simone et al., 2014a) regression models, or gaussian process regression (GPR) (Schiemer et al., 2023), just to name a few. Further explanations of chemometric methods can be found in published literature (Wold et al., 2001; Chen et al., 2007; Bro and Smilde, 2014; Acquarelli et al., 2017). Additional preprocessing of Raman spectra (Bocklitz et al., 2011) improves the chemometric analysis and helps to reduce the complexity of the data set, extract essential information from spectral data, and remove spectral noise or unwanted experimental disturbances, particularly in situations involving multiple species and interferential effects. Crystallization processes of mostly chemical substances were monitored spectroscopically in the past using PCA (Simone et al., 2016), principal component artificial neural networks (PC-ANN) (Saleemi et al., 2012b), principal component regression (PCR) and PLS (Simone et al., 2014a; Lin et al., 2020; Jul-Jørgensen et al., 2024), or multiple linear regression (MLR) (Mercado et al., 2008). As regards PAT, there have been numerous attempts to monitor crystallization of chemical compounds in pure (Bakar et al., 2009; Simone and Nagy, 2015) or relatively pure mixtures (Wang et al., 2000; Togkalidou et al., 2002; Jul-Jørgensen et al., 2024). With respect to biologics, PAT studies investigating the crystallization process of the benchmark crystallization protein lysozyme in model protein solutions (Mercado et al., 2008; Tian et al., 2023) or of small, biological molecules with low levels of impurities (Simone et al., 2016) were discussed. To the best of the authors’ knowledge, however, no research has been conducted into using PAT for protein crystallization to capture larger biological targets in heterogeneous, complex mixtures, i.e., clarified lysate. Real-time monitoring would extend our understanding of protein crystallization in complex solutions and facilitate process control.

To find a suitable PAT set-up for protein crystallization in a heterogeneous mixture, this research project investigates different spectroscopic methods, their limitations, and possible implementation for the application to crystalline slurries. The molecule of interest is the enzyme *Lactobacillus kefir* alcohol dehydrogenase (*Lk*ADH) which is crystallized from clarified lysate in a stirred vessel on lab-scale. To increase the variety of the recorded data sets, five batch experiments are conducted with varying crystallization conditions in terms of precipitant concentration, initial absorption value of the clarified lysate, and changes in the lysis protocol. An *in situ* Raman probe is immersed directly into the crystallization vessel and records in-line spectra which are processed with chemometric methods to predict product characteristics, e.g., target molecule concentration in the liquid phase. An analytical bypass of the crystallization vessel is realized with a cross-flow filtration (CFF)-based set-up to facilitate the use of particle-sensitive analytics, i.e., UV/Vis spectroscopy with a VP flow cell. Microscopic imaging, *Lk*ADH and host cell protein (HCP) quantification of off-line samples - and optionally redissolved crystals - assist in developing a comprehensive process understanding of the crystallization process in complex lysate. In short, the results demonstrate how a protein crystallization PAT can be realized for process and product characteristics in a heterogeneous, complex solution where multiple phases are present.

## 2 Material and methods

### 2.1 Experiment buffer and protein preparation

All chemicals were purchased from Merck KGaA Darmstadt, DE, unless otherwise stated. The buffer solutions were prepared at room temperature with ultrapure water (PURELAB Ultra, ELGA LabWater, Lane End, High Wycombe, U.K.), pH-adjusted with 32% hydrochloric acid (HCl) or 4 M sodium hydroxide (NaOH), and filtered using a 0.2 µm cellulose acetate (CA) membrane filter (Sartorius Stedim Biotech GmbH, Göttingen, DE).

The *Lk*ADH protein (wildtype (WT); protein data bank (PDB) ID: 7P36) was produced with *E. coli* (*Escherichia coli*) BL21(DE3) in a fed-batch process in 1.5 L stirred-tank bioreactors (DASGIP, Eppendorf GmbH, Hamburg, DE) as described in Schmideder et al. (2016). The process is divided in three consecutive phases at pH 7.0: batch phase (5.0 g L^−1^ glucose, 4 h at 37 °C), exponential feeding phase (growth rate 0.15 h^−1^, 18 h at 37 °C), and protein production phase (500 µM isopropyl β-d-1-thiogalactopyranoside (IPTG), 3.0 g L^−1^ h^−1^ glucose, 48 h at 30 °C). The harvested *E. coli* cells were kindly provided by the research group of Prof. Weuster-Botz. The cell pellets were further processed as described in Walla et al. (2021), with variations listed in the Supplementary Table S1. The cell pellets were sonified in an ice bath by the sonifier SFX550 (Branson Ultrasonic Corporation, Danbury, US-CT, tapered microtip 101-148-062, 70% amplitude, 50% pulse, 40 s) twice to three times and with 3 min breaks between each cycle. Cell debris was removed from the supernatant by centrifugation at 4 °C with 17,418 rcf for 1 h and by filtration with a glass fiber, a 0.45 µm, and a 0.2 µm CA syringe filter (Sartorius Stedim Biotech GmbH, Göttingen, DE). After dialysis (SnakeSkin^TM^ dialysis tube, ID 34 mm, molecular weight cut-off (MWCO) of 3.5 kDa, Thermo Fisher Scientific, Inc., Waltham, MA), the filtered supernatant to the protein buffer (20 mM 4-2-hydroxyethyl-1-piperazineethanesulfonic acid (HEPES), 1 mM magnesium chloride (MgCl_2_) at pH 7.0), the initial absorption value *A*_initial_ at 280 nm of the clarified lysate was adjusted with protein buffer according to Table 1 using a NanoDrop^TM^ 2000 spectrometer (Thermo Fisher Scientific, Inc.).

**TABLE 1:** Crystallization conditions, HCP content, and crystal yield: The variations of the crystallization conditions covered the initial absorption at 280 nm *A*_280 nm_, *c*_PEG_, and the number of lysis cycles. The experiments were performed with or without the analytical bypass. The HCP content of the first sample is compared with the content of the washed and redissolved crystals. To account for differences in dilution, the HCP content was normalized to the target molecule concentration. The crystal yield was estimated by the ratio of the initial to equilibrium *Lk*ADH concentrations in the supernatant and derived from the IMAC analysis (see Section 2.3.3).

exp	Crystallization conditions			Bypass	HCP removal			Crystal yield in %
	*A*_280 nm_ in AU/cm	*c*_PEG_ in g/L	Number of lysis cycles		*c*_HCP, initial_ in μg/L	*c*_HCP, RD_ in μg/L	HCP reduction factor	
Exp1	10.0	100	3	w/	53,460	n.d	n.d	46.6
Exp2	10.3	125	2	w/	27,412	358	77	77.9
Exp3	10.4	150	2	w/	42,438	n.d	n.d	77.9
Exp4	14.3	100	2	w/	38,644	n.d	n.d	27.9
Exp5	10.3	125	2	w/o	42,982	437	98	76.4

Abbreviations - HCP: host cell protein; n.d: not determined; PEG: polyethylene glycol; RD: redissolution; w/: with; w/o: without.

The crystallization buffer was a 100 mM tris(hydroxymethyl)aminomethane (Tris), 50 mM MgCl_2_ buffer with a varying polyethylene glycol monomethyl ether 550 (PEG MME 550) concentration depending on the experiment (see Table 1). The redissolution (RD) buffer was a 20 mM HEPES, 2 M MgCl_2_ buffer at pH 7.0. The required buffers for the immobilized metal ion affinity chromatography (IMAC) analysis contained 50 mM phosphate, 500 mM sodium chloride (NaCl), and 20 mM imidazole for equilibration or 200 mM imidazole for elution (both pH 7.0).

### 2.2 Protein crystallization experiment

The batch crystallization process was initiated in a 300 mL jacketed glass vessel (CG-1929-X11) equipped with an overhead stirrer of 80 rpm speed (CG-2024-10, both provided by Chemglass Life Sciences, Vineland, US-NJ, anchor style stir paddle). 150 mL clarified lysate were fed into the vessel and 150 mL crystallization buffer were added manually. The crystallization conditions varied according to Table 1 for the five conducted experiments Exp1 - Exp5. After 30–90 min, the vessel content was removed and centrifuged (in two 150 mL vessels for 15 min at 3,225 rcf) to remove initial HCP and nucleic acid precipitate which was caused by the addition of the crystallization buffer. The supernatant was placed in the glass vessel, the experiment continued, and the target molecule crystallized after the centrifugation step.

### 2.3 Analytics

**FIGURE 1:**
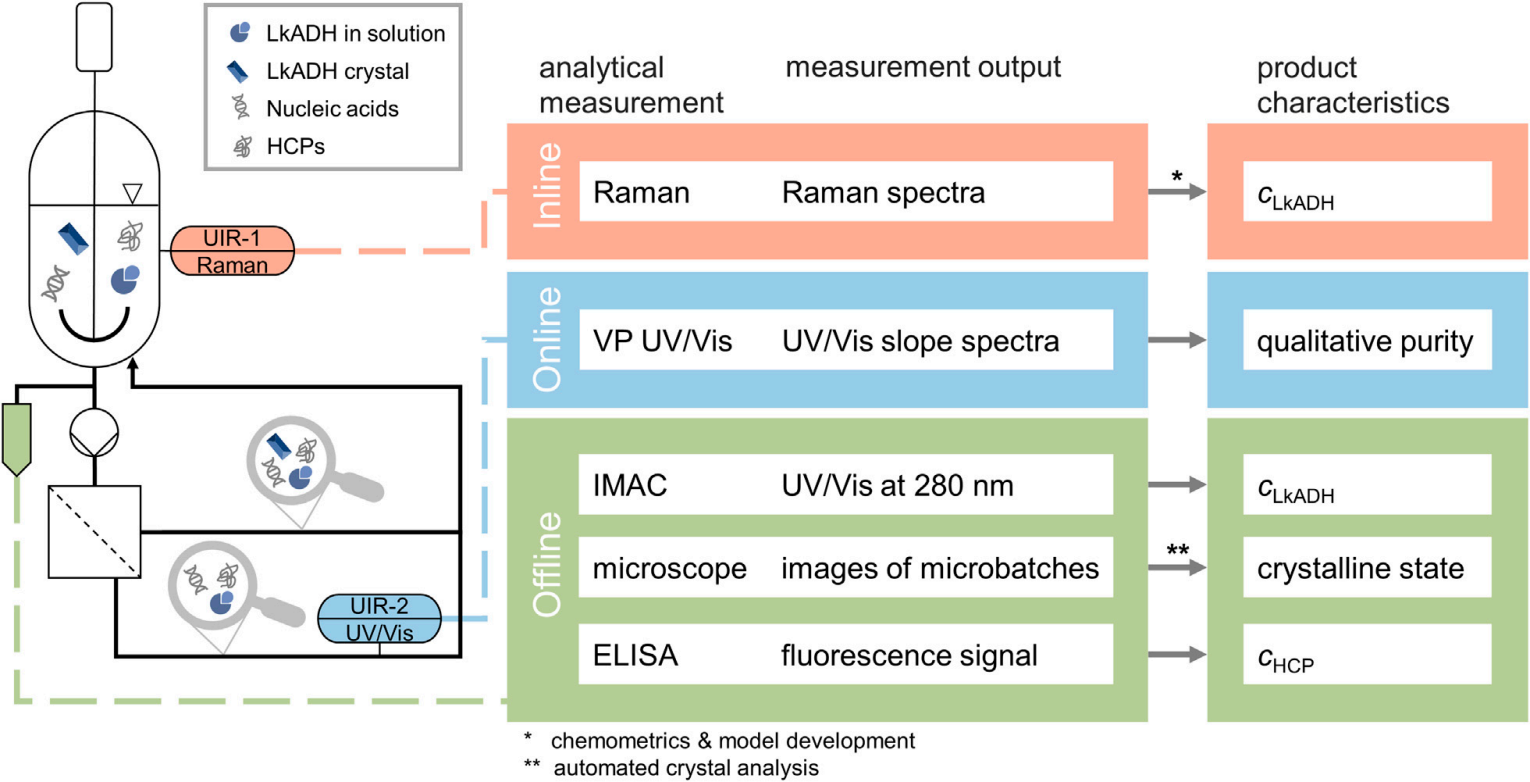
Experimental and analytical set-up of the protein crystallization experiments as a scheme. The desired product characteristics are listed on the right together with the respective analytical measurement method and output. The vessel contains the clarified lysate with the HCPs, nucleic acids, the molecule of target *Lk*ADH, and later during the process *Lk*ADH crystals while the installed Raman probe records in-line spectra. The CFF-based set-up facilitates the solid-liquid separation, thus enabling on-line VP UV/Vis measurement in the permeate stream. Both the retentate and permeate streams are directed back to the vessel. All off-line samples are analyzed with IMAC and microscopic imaging. Selected samples are further analyzed with automated ELISA to determine the HCP content.

The following section describes the PAT set-up to monitor protein crystallization using in-line Raman spectroscopy, a filtration-based on-line UV/Vis set-up and off-line samples. The set-up of the different analytics is visualized and listed in Figure 1.

#### 2.3.1 In-line Raman spectroscopy

To monitor the crystallization process by in-line Raman spectroscopy, a MarqMetrix Bioreactor Ballprobe (MarqMetrix^®^, Seattle, US-WA) was immersed into the crystallization suspension and connected to a HyperFlux^TM^ PRO Plus 785 Raman analyzer with Spectralsoft 3.3.600.1 (Tornado Spectral Systems, Mississauga, CA). The measurement was performed with a laser power of 495 mW at the laser wavelength of 785 nm and an exposure time of 8,553 ms, averaging 15 spectra every 12 min in the Raman shift range 200–3,300 cm^−1^.

#### 2.3.2 Analytical bypass and on-line analytics

As an analytical bypass, a CFF-based set-up was installed to separate the solid precipitate and crystals from the supernatant, facilitate the implementation of particle-sensitive devices, and prevent clogging by solid particles.

A KrosFlo Research KRIIi CFF system was equipped with an automatic back-pressure valve, pressure transducers (all Spectrum Labs, Rancho Dominguez, US-CA), and CFF membrane (modified polyethersulfone (mPES), 0.2 µm pore size, 13 cm^2^ surface area, C02-P20U-10-N, Spectrum Laboratories, Inc., Rancho Dominguez, US-CA). The feed flow rate and the desired transmembrane pressure (TMP) were set to 20 mL min^−1^ and 0.05 bar, respectively. Due to potential tube blockage and damage to the devices, the bypass was switched off over night and the bypass suspension was pumped into the crystallization vessel. Subsequently, the bypass and the membrane were cleaned with water at 40 °C. The liquid flow meter SLS-1500 (Sensirion AG, Stäfa, CH) was installed in the permeate plug in the analytical bypass and recorded the permeate flow averaged over a time range of 5 s. As UV/Vis spectroscopy is sensitive to larger particles and light scattering, the on-line FlowVPE flow cell (C Technologies, Inc., Bridgewater, US-NJ) with a Cary 60 spectrometer (Agilent Technologies, Inc., Santa Clara, US-CA) was implemented in the analytical bypass to measure the UV/Vis absorption slope spectra in the permeate flow between 220 and 400 nm.

#### 2.3.3 Off-line analytics

Off-line samples were taken during the crystallization process through an injection plug (Fresenius Kabi AG, Bad Homburg, DE) in the feed flow. For visual crystal detection, suspension samples were 10 times diluted to prevent proceeding crystallization. For the supernatant analysis, the samples were centrifuged (2 min, 12,000 rcf) and the diluted supernatants (2 times) for IMAC and enzyme-linked immunosorbent assay (ELISA) were stored at −20°C until analysis. Grown crystals were redissolved by removal of the supernatant after centrifugation, washing with protein buffer, a second centrifugation step, redissolving in RD buffer, and a third centrifugation step. The centrifugation procedure is described above.

For visual inspection of the crystalline suspension, 24 µL-quadruplicates of the undiluted and 10 times diluted suspension were placed in a MCR Under Oil Crystallization Plate (Hampton Research, Aliso Viejo, CA), sealed with a transparent foil (Shurtape Technologies, LLC, Hickory, US-NC), and imaged using a tempered microscopic system (RI 54, FORMULATRIX LLC, Bedford, US-MA, T 1000 mytron Bio-und Solartechnik GmbH, Heiligenstadt, DE) at 20°C . As the sampling time for the microscopic imaging was less than 20 min and short compared to the protein crystallization time, crystal nucleation or growth in the static micro-batch samples is not expected. Next to manual, visual inspection, a machine learning (ML) model based on augmented, synthetic images of crystals (Bischoff et al., 2022) counted and measured the crystal height and width to detect crystals objectively and automatically. Images were treated as outliers and not included in the analysis when they were out of focus or showed large bubbles. Using the model as a basis, the following small adaptations were applied to adjust the detection method to the setup used in the presented experiments. The border due to the circular well geometry was removed from the microscope images by cropping the image to the central region with a size of 1400 × 1200 pixels. Furthermore, large-area false positive detections could be eliminated by applying a threshold for the maximum crystal size of 10^3^ px^2^. The confidence threshold for accepted detections was set to 0.2. Finally, inference was performed on a GTX 1080 GPU.

The IMAC analysis was performed as a reference for the *Lk*ADH concentration *c*_LkADH_ as the target molecule contained a His-tag. A TSKgel^®^ Chelate-5PW column (Tosoh Corporation, Shiba, JA) with a pre-column filter (0.2 µm, OPTI-SOLV EXP, Supleco^TM^, Bellefonte, US-PA) was installed in a Dionex Ultimate 3000 RS high-performance liquid chromatography (HPLC) system (Thermo Fisher Scientific, Inc.) equipped with a diode array detector. The supernatant samples were thawed and filtered with an AcroPrep^TM^ Advance filter plate (3.0 µm glass fiber/0.2 µm Supor^®^ membrane, Pall Corporation, Port Washington, NY). Either 20 µL supernatant samples or 40 µL of the redissolved crystals (filtered as above) were analyzed with a two-step elution protocol using 100 mM and 200 mM imidazole to elute loosely bound impurities and the target molecule, respectively (see Supplementary Material S1). The absorption was used to quantify *Lk*ADH. The elution peak absorption and the extinction coefficient 0.8596 AU*L/(g*cm) (derived from the web-tool ProtParam (Gasteiger et al., 2005)) at 280 nm were used to quantify *Lk*ADH.

The HCP concentration of selected supernatant and redissolved crystal samples was determined using the Gyrolab XPlore station with its software Gyrolab Control 7.0.3.133 (Gyros Protein Technologies AB, Uppsala, SE) following the manufacturer’s protocol and used to evaluate the HCP removal by *Lk*ADH crystallization and RD. Due to sampling errors during experimental handling, crystal pellets could only be redissolved and HCP-analyzed for Exp2 and Exp5.

### 2.4 Data analysis

Data analysis, including spectral preprocessing, model calibration, and data plotting, was performed in MATLAB, R2019b (The MathWorks, Inc., Natick, MA). To contrast different sampling approaches, the Kennard-Stone (KS) data split algorithm (Kennard and Stone, 1969; Westad and Marini, 2022) and a manual data split approach were tested for model validation. Spectral preprocessing for Raman spectra was implemented to highlight significant spectral features which can then be correlated to the desired process parameter to enable PAT.

The *mdatools* toolbox (Kucheryavskiy, 2020) was applied to the Raman spectra using the baseline correction with asymmetric least squares (smoothness 10,000, penalty value 0.01). The spectra were treated with the Savitzky-Golay (SG) filter (Savitzky and Golay, 1964, KS data split: window size 29, second derivative, manual data split: window size 17, first derivative) and cut to the Raman wavenumber regions 300–490, 750–1,040, 1,210–1,320, and 2,800–3,000 cm^−1^.

The Raman spectra of all experiments were baseline-corrected and analyzed with the unsupervised learning method PCA to reduce the dimensionality of the data set and visualize correlation between the spectra and the crystallization process.

The optimal parameters for preprocessing, namely, window size and SG filter derivative, as well as model calibration, i.e., number of latent variables, were optimized using a genetic algorithm (GA). For details on the methodology, the authors refer to Andris et al. (2018). The PLS regression model, as a supervised learning method, was employed to predict the concentration of the target molecule in the supernatant. As a first step, the Raman spectra closest to the sampling time were selected and grouped into a calibration and a validation data subset consisting of 29 and five experimental samples, respectively. Only Raman spectra and off-line samples after the centrifugation step were included as the removal of precipitated impurities considerably affected the model calibration. To assess the impact of two distinct data splitting methods on the model prediction, the samples were initially divided using the KS algorithm which selects a representative data subset from a larger data set. As an alternative, manual data split approach, the samples of Exp5 were selected as the external validation set to examine the model predictability for new experiments and batch-to-batch variations. Finally, the *Lk*ADH concentrations obtained from the IMAC analysis were regressed against the preprocessed spectra of the calibration subset by leave-one-out cross-validation. The model calibration procedure with KS or manual data splitting resulted in eight or 10 latent variables, respectively.

## 3 Results

### 3.1 Off-line: Image analysis, *Lk*ADH and HCP quantification

In this project, the crystal yield was estimated by the decrease from the initial to the equilibrium concentration of the target molecule and can be used to evaluate and compare processes. Furthermore, the HCP concentration of the initial solution and the redissolved crystals was determined and normalized to the target molecule concentration providing information on the purity and HCP removal for this process. These values and the experimental conditions are listed in Table 1 and demonstrate a 77-fold and 98-fold HCP removal in Exp2 and Exp5 while achieving a yield of 77.9% and 76.4%, respectively.

**FIGURE 2:**
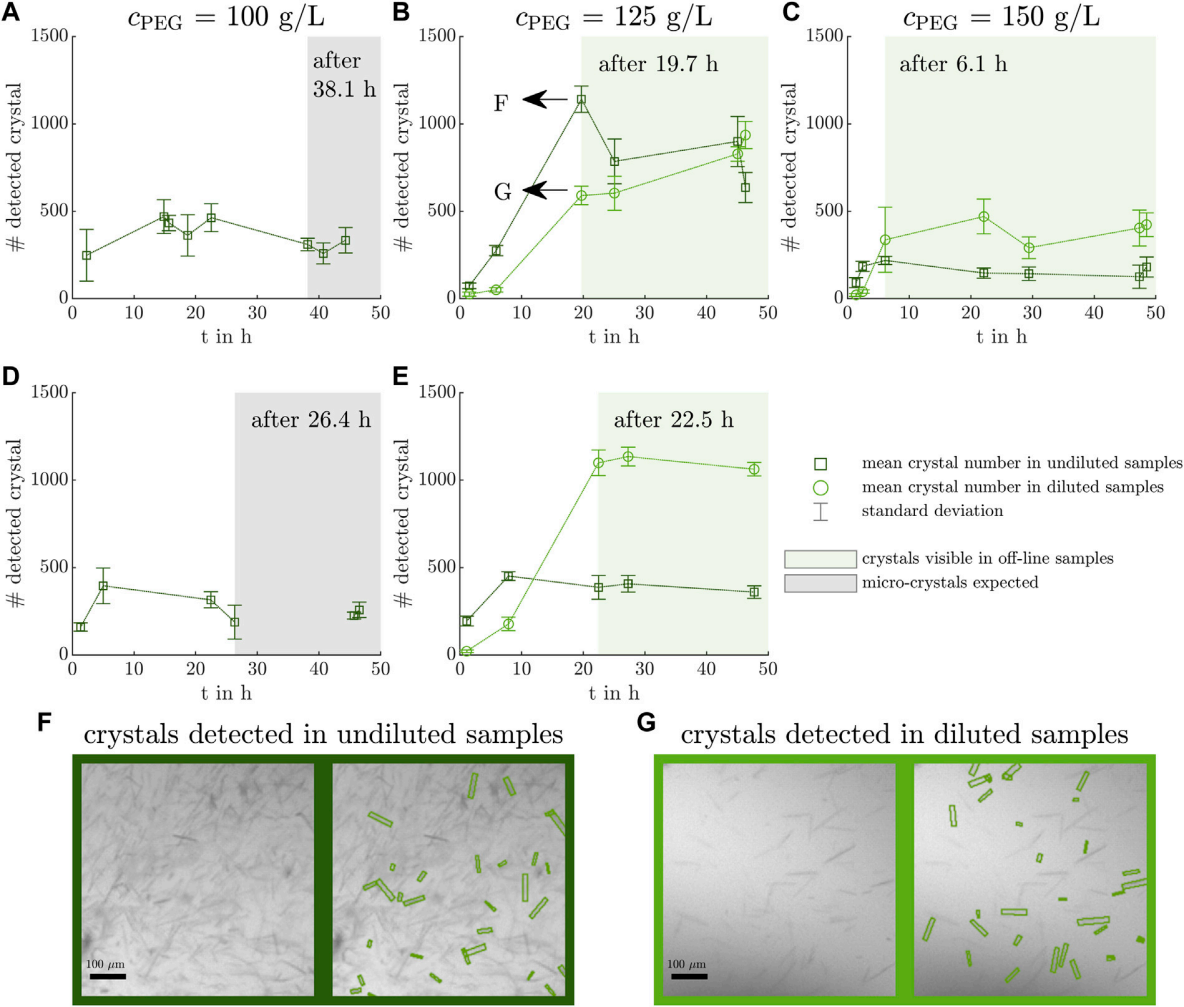
Counted crystals in microscopic images of off-line samples. The microscopic images of off-line samples are analyzed with a ML-based image analysis tool (Bischoff et al., 2022) counting the crystals and providing information on the crystal geometry (see Supplementary Material 2.3). The mean crystal count per imaged well and its standard deviation of undiluted and diluted off-line samples are visualized over the experimental time with dark green squares and light green circles with dotted lines to guide the eye and with their respective error bars. The off-line samples with micro-crystals present are shaded in gray as they are difficult to detect due to the image resolution **(A, D)**. The off-line samples showing larger crystals are shaded in a light green box **(B, C, E)**. Exemplary, the results of the automated image detection are shown for an undiluted **(F)** and a diluted **(G)** off-line sample of Exp2 after 19.7 h. The crystallization conditions for the experiments Exp1 - Exp5 **(A–E)** are listed in Table 1.

The mean counts of detected crystals per off-line sample of the five conducted experiments are depicted over time with their standard deviations in Figures 2A–E. The light green and gray shaded areas indicate off-line samples in which crystals of larger size were visible and micro-crystals were assumed to exist as the latter were difficult to detect due to the image resolution. For Exp1 and Exp4, the mean counts of detected crystals fluctuate between 200 and 500  whereas Exp2 and Exp5 start with detected crystal counts less of than 100 and increase to values above 1,000 after 20 h. The crystal count of Exp3 rises to values above 400 after 6 h. A trend towards lower crystal detection points in time with increasing polyethylene glycol (PEG) concentration is visible from left to right. The numbers of detected crystals were largest in Exp2 and Exp5, which also demonstrated slightly higher crystal heights (see Supplementary Figure S4). In Exp1 and Exp4, the automated image analysis detected crystals in undiluted samples in Figures 2A,D. The manual, visual inspection indicated micro-crystals in those experiments after 38 h and 26 h, respectively. Therefore, the mean count of detected crystals determined before the specified time points may be an artifact of image noise which is falsely detected as crystals.

In two exemplary microscopic images of the same off-line sample of Exp2 after 19.7 h, the ML-based model-detected crystals are highlighted in Figures 2F, G. The microscopic images differed in the dilution factor to account for high crystal densities and reduce overlapping crystals. As not all visible crystals are highlighted in Figures 2F,G, the image analysis tool is used in addition to the manual inspection as an objective, qualitative tool to narrow down the samples with crystals present and to provide insight into the crystal geometry.

### 3.2 On-line: Analytical bypass and UV/Vis spectroscopy

**FIGURE 3:**
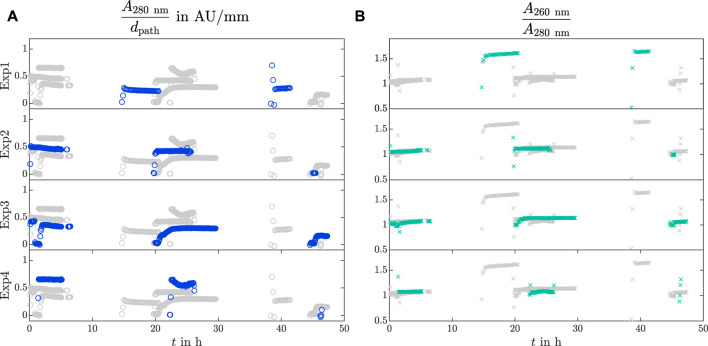
UV/Vis analysis in the analytical bypass. The recorded VP UV/Vis slope $\frac{A_{280\text{nm}}}{d_{\text{path}}}$ **(A)** and the $\frac{A_{260\text{nm}}}{A_{280\text{nm}}}$ **(B)** ratio of Exp1 - Exp4 are shown over time by blue circles and turquoise crosses, respectively. For comparison and clearer visualization, each row emphasizes one experiment and shows the other three experiments in light gray.

The analytical bypass was installed to make particle-sensitive analytics feasible in crystallization processes. The results of the VP UV/Vis spectroscopy are depicted in Figure 3 over time for Exp1 to Exp4, as Exp5 did not have a bypass installed (see Table 1). Each row emphasizes the absorption data of one experiment with colored markers. The other experiments are shown in gray markers for better comparison. The absorption at 280 nm *A*_280nm_ is derived with respect to the path length *d*_path_ and visualized by blue circles over time *t* as $\frac{A_{280nm}}{d_{\text{path}}}$ in Figure 3A. The ratio between the absorption values at 260 nm and 280 nm as $\frac{A_{260nm}}{A_{280nm}}$ is depicted by turquoise crosses over time in Figure 3B. As the bypass was only operated during the day, data is lacking during nighttime.

The absorption slope $\frac{A_{280nm}}{d_{\text{path}}}$ can indicate changes in the concentration of UV/Vis absorbing material in the supernatant. After switching on the analytical bypass, the absorption slope data stabilized within 0.5 to 4 h to a constant value. On the first day within the first 7 h, the absorption slope decreased in Exp2 and Exp3 whereas Exp4 did not show decreasing absorption values. The absorption data of Exp1 were not recorded. The absorption slopes of Exp1 to Exp3 stabilized after 2–4 h on the second day. The third day showed stable values in Exp1, Exp2, and Exp3 whereas the absorption values of Exp4 did not stabilize due to tube blockage.

The absorption ratio $\frac{A_{260nm}}{A_{280nm}}$ , as an indicator for nucleic acid and protein content, stabilized to values around one in Exp2 to Exp4. The highest $\frac{A_{260nm}}{A_{280nm}}$ ratios of 1.15 were achieved on the second day. Analogous to the absorption slope at 280 nm in Figure 3A, the ratios stabilized after shorter times on the second and third days after switching on the analytical bypass. The ratio in Exp1 was around 1.6. This was the only experiment which included three lysis cycles.

The TMP over the CFF membrane and the flow rate of the permeate stream can help to evaluate the reliability of the on-line sensor implemented in the analytical bypass as the sensor can only measure reliably if the solution in the bypass represents the current particle-free vessel content. Information on the TMP over the CFF membrane and the flow rate of the permeate stream is enclosed in the Supplementary Material S2A, B.

### 3.3 In-line: Raman spectroscopy and exploratory analysis

**FIGURE 4:**
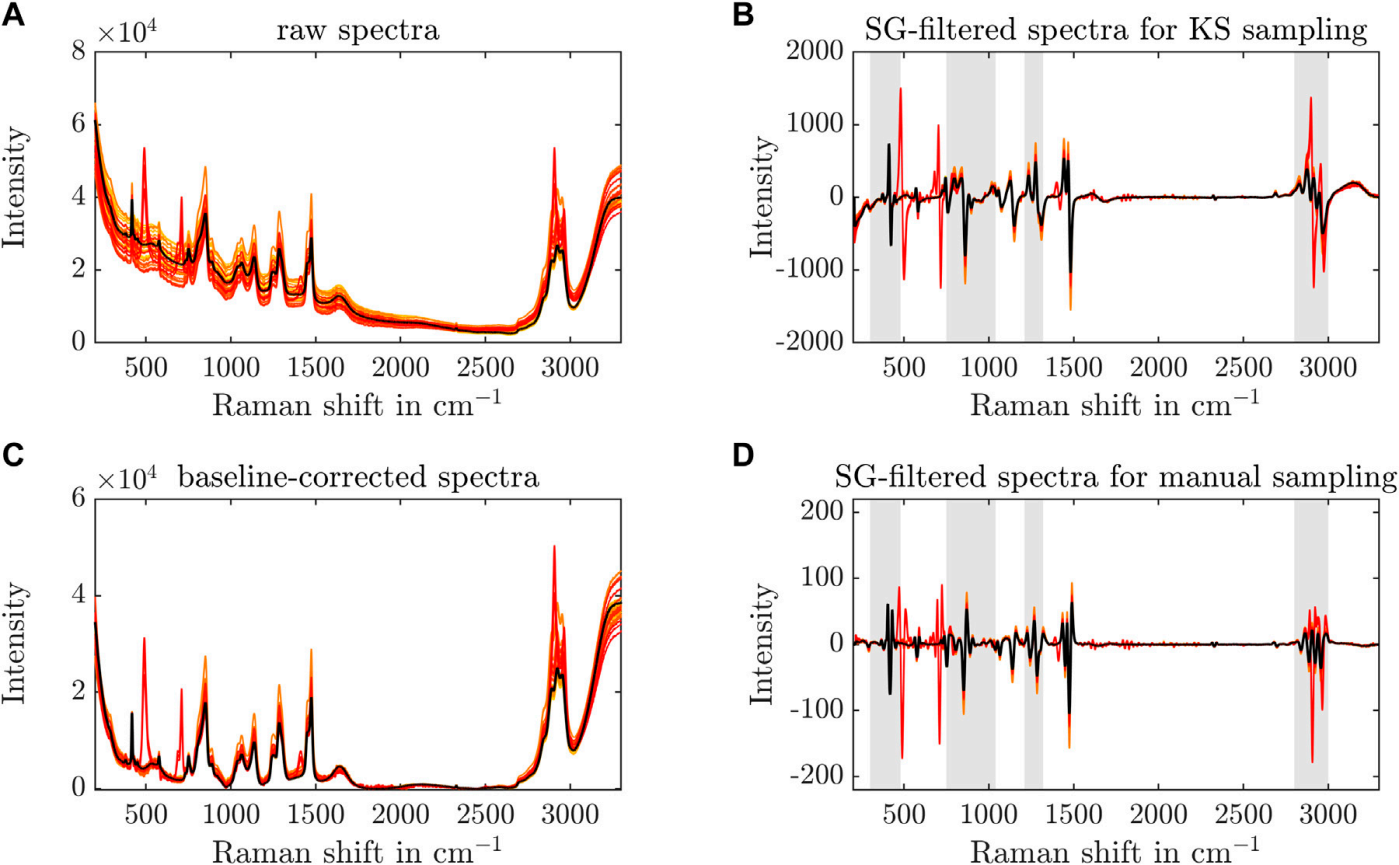
Preprocessing of Raman spectra. The raw Raman spectra, used for regression modeling, are shown over the recorded wavenumber range from yellow to red to visualize the different samples of all experiments **(A)**. The black line represents one specific spectrum to better visualize the preprocessing effects. Preprocessing techniques, such as the baseline correction **(C)** and the SG filter, are applied to the spectra with a KS **(B)** or manual data split **(D)** to enhance spectral differences. The gray boxes in **(C, D)** depict the Raman shift ranges that are used for the PLS model development.

To monitor the stirred-batch crystallization process, a Raman probe was installed in the vessel to record in-line spectra over time. Spectral preprocessing is advised to enhance spectral differences and remove baseline drifts, background signals, or detector noise. Generally, several techniques are tested to find a matching set of preprocessing steps in most cases, e.g., baseline correction, background subtraction, normalization, centering. Figure 4 shows the effects of the preprocessing steps on the spectra later used for the regression model. All recorded spectra are preprocessed and visualized in yellow to red color with one arbitrary spectrum in black to better visualize the preprocessing effects on one exemplary spectrum. The raw spectra (see Figure 4A) are baseline-corrected (see Figure 4B) and treated with a SG filter - second derivative for the KS or first derivative for the manual data split (see Figures 4C,D). The selected wavenumber ranges for the PLS regression model development are illustrated with gray shaded boxes in (see Figures 4C,D). The selection of preprocessing steps reduces the baseline drift visible in Figures 4A,B, aligns the spectra, and helps to increase spectral differences. The calculation of the derivatives emphasizes peak shifts in the examined spectra near 790 cm^−1^, 1,260 cm^−1^, or 2,970 cm^−1^. Beforehand, different normalization, derivative, and baseline correction methods were tested, but did not improve the interpretability of the data. To demonstrate the preprocessing effects on the experimental data, a zoom into the selected wavenumber regions of Exp3 is included in the Supplementary Material S6 as an example.

**FIGURE 5:**
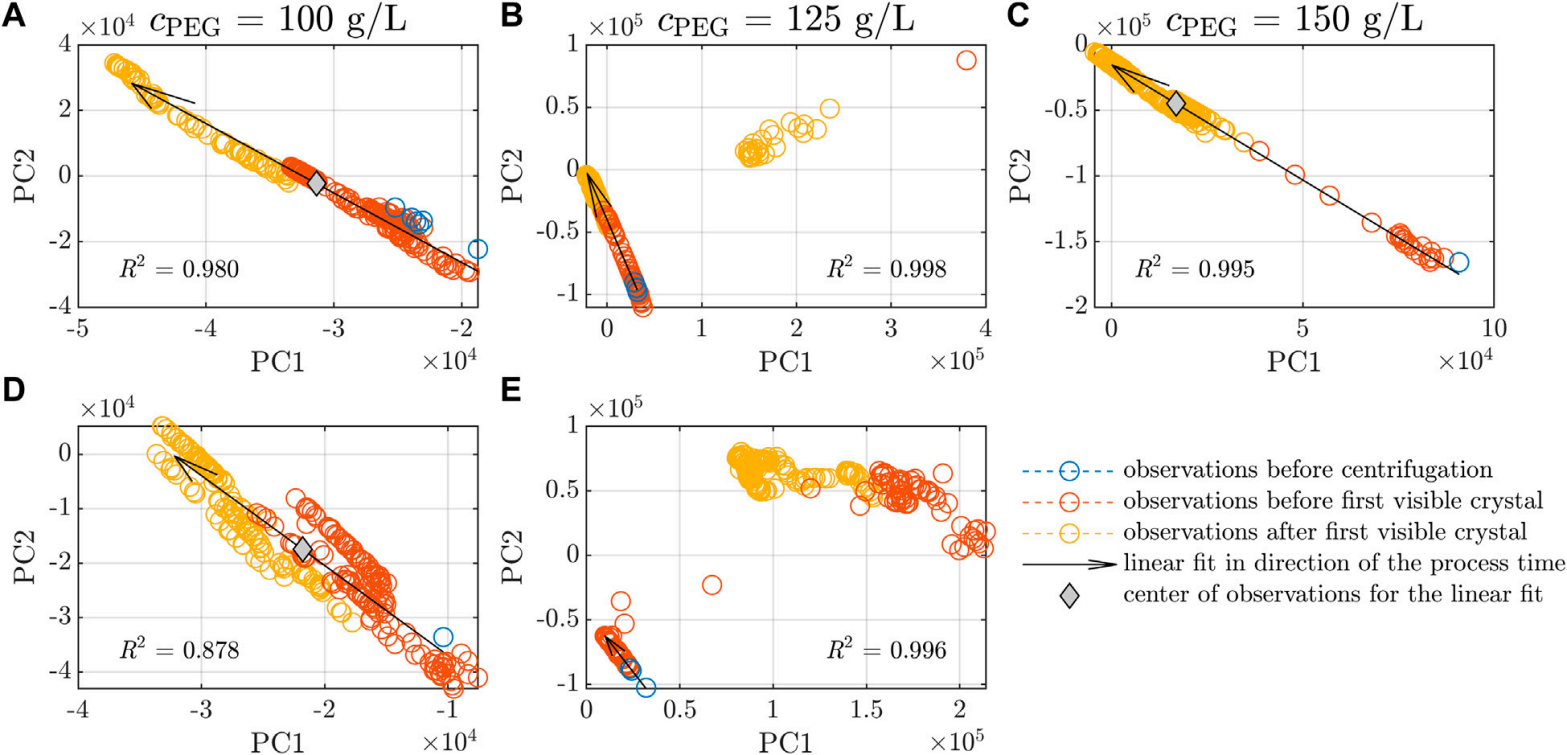
PCA scores of Raman spectra. The scores of the first & second PCs are identified as PC1 and PC2 and are shown for the five experiments **(A–E**, see Table 1). The observations of each experiment are classified by investigation of the off-line microscopic images. Observations before the initial centrifugation step, after the centrifugation step until the first, visual occurrence of crystals and after the first detected crystals are shown in blue, orange, and yellow, respectively. Linear fits of all data in **(A, C, D)** and the main trend in **(B, E)** are visualized by black arrows. The arrow direction shows the observations *versus* the process time. The coefficients of determination *R* ^2^ of the linear fits are included. The gray diamonds symbolize the center of the data in the experiments **(A, C, D)**.

A PCA analysis of a large data set can help visualize trends and cluster observations in groups. It was applied to the preprocessed Raman data of all five experiments in this study using the whole spectral range (200–3,300 cm^−1^). Figure 5 depicts the principal component PC2 over PC1 of the PCA, with each subfigure depicting the spectra of one experiment. The colors blue, orange, and yellow represent observations before the centrifugation step (see section 2.2) before and after the first crystals were detected in the microscopic images of the samples examined off-line. Linear fits and the center of the observation data are marked by black arrows and gray diamonds, respectively. The PCA loadings can be found in the Supplementary Material S7. With passing time of the crystallization experiment, PC1 decreases whereas PC2 increases as indicated by the arrows. The arrows demonstrate a comparable slope when all observations are visualized in one diagram (figure not shown). The experiments Exp2 and Exp5 show two clusters. The left clusters follow the direction of the arrows. The right clusters do not follow this direction and could be traced back to the irregular peaks which can be seen in Figure 4 near 493 cm^−1^, 708 cm^−1^, 1,410 cm^−1^, 2,909 cm^−1^, and 2,970 cm^−1^. For the linear trend and the calculation of the coefficient of determination *R* ^2^, the data of the left cluster only were included. Among the five experiments, the observations of Exp4 stand out as they follow the direction of the linear fit, but are more widely scattered as indicated by the lower *R* ^2^ of 0.878. Exp3 observations move from the lower right to the upper left corner of Figure 5C. Before crystallization was detected in the off-line samples, observations were located at the end of the arrows whereas the observations after crystallization were located near the tip of the arrows. In contrast to Exp1 and Exp4 (see Figures 5A,D), the center of observations for Exp3 was found at lower PC1 and higher PC2 values, indicating a faster change in the preprocessed Raman spectra. Further inspection of the preprocessed spectra over time showed that the changes before and after crystallization were reflected by gradually reduced peak heights in the spectra (data not shown).

### 3.4 PLS model development and application to protein concentration monitoring

For the development of a PLS model, the preprocessed spectra nearest to the sampling time are regressed on the off-line concentration *c*_LkADH_ from the IMAC-analyzed samples. Then, the developed model is applied to all spectra which were recorded during the batch experiments to assess its potential to monitor real-time concentrations of the target molecule.

**FIGURE 6:**
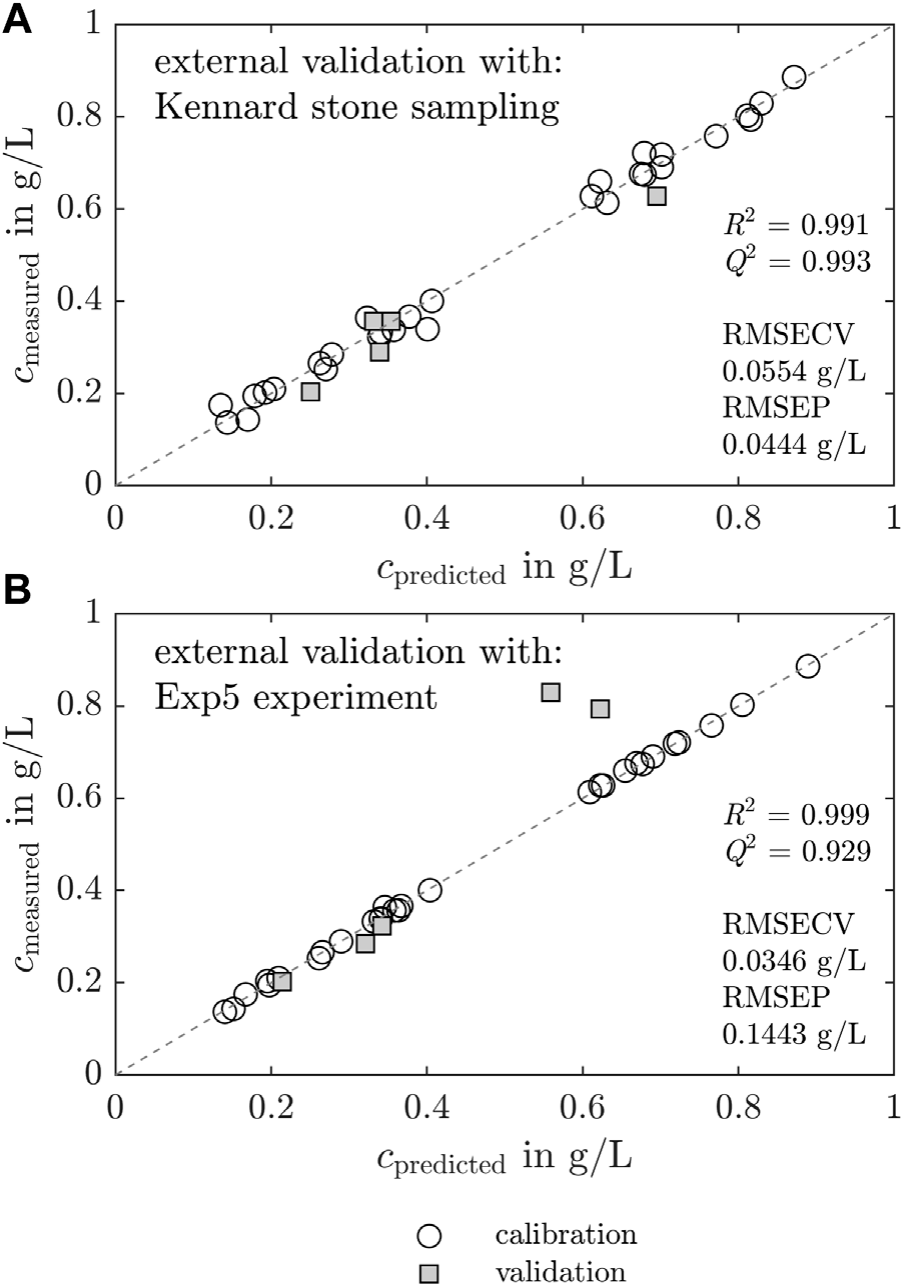
Chemometric regression model based on Raman spectra and effects of validation sampling techniques. The preprocessed Raman spectra are regressed on the IMAC derived *Lk*ADH concentration with PLS models. Two models differing in the choice of the validation data set are compared. The white circles, gray squares, and dashed line represent the calibration, validation data, and theoretical values, respectively. First, the measured concentrations are visualized *versus* the model-predicted concentrations in **(A)** for a model with KS data split. Analogous plots are shown in **(B)** for a model where Exp5 was chosen manually as the validation data set. High *R* ^2^ and *Q* ^2^, and low *RMSECV* and *RMSEP* values indicate an applicable model.

Figure 6 shows the results of two separately calculated PLS models which differed only in the choice of the data split for the external validation. The KS algorithm chooses the external validation samples according to the uniform distribution within the data set. For the model with a KS data split, the measured concentrations of the target molecule are shown *versus* the predicted concentrations in Figure 6A. The white circles and gray squares represent the calibration and external validation set, respectively. For the second model, Exp5 was selected manually as the external validation set to evaluate the PLS model transferability to new experiments. The measured concentrations of the second model with the manual data split are illustrated *versus* the predicted concentrations in Figure 6B.

For both models, the calibration data fit the dashed line. Hence, the models are suitable and the preprocessed spectra and *c*_LkADH_ correlate in the calibration data set. The external validation data set chosen with a KS data split fits the ideal line well, with maximum discrepancies of 0.07 g L^−1^ (see Figure 6A). The external data of the PLS model with the manual data split fits the ideal, dashed line below concentrations of 0.4 g L^−1^ well, but two outliers are visible at higher concentrations around 0.8 g L^−1^ with maximum deviations of 0.27 g L^−1^. PLS model metrics, i.e., the coefficient of determination (*R* ^2^), predictive relevance (*Q* ^2^), root mean squared error of cross-validation (*RMSECV*), and root mean squared error of prediction (*RMSEP*), are added in Figures 6A,B.

**FIGURE 7:**
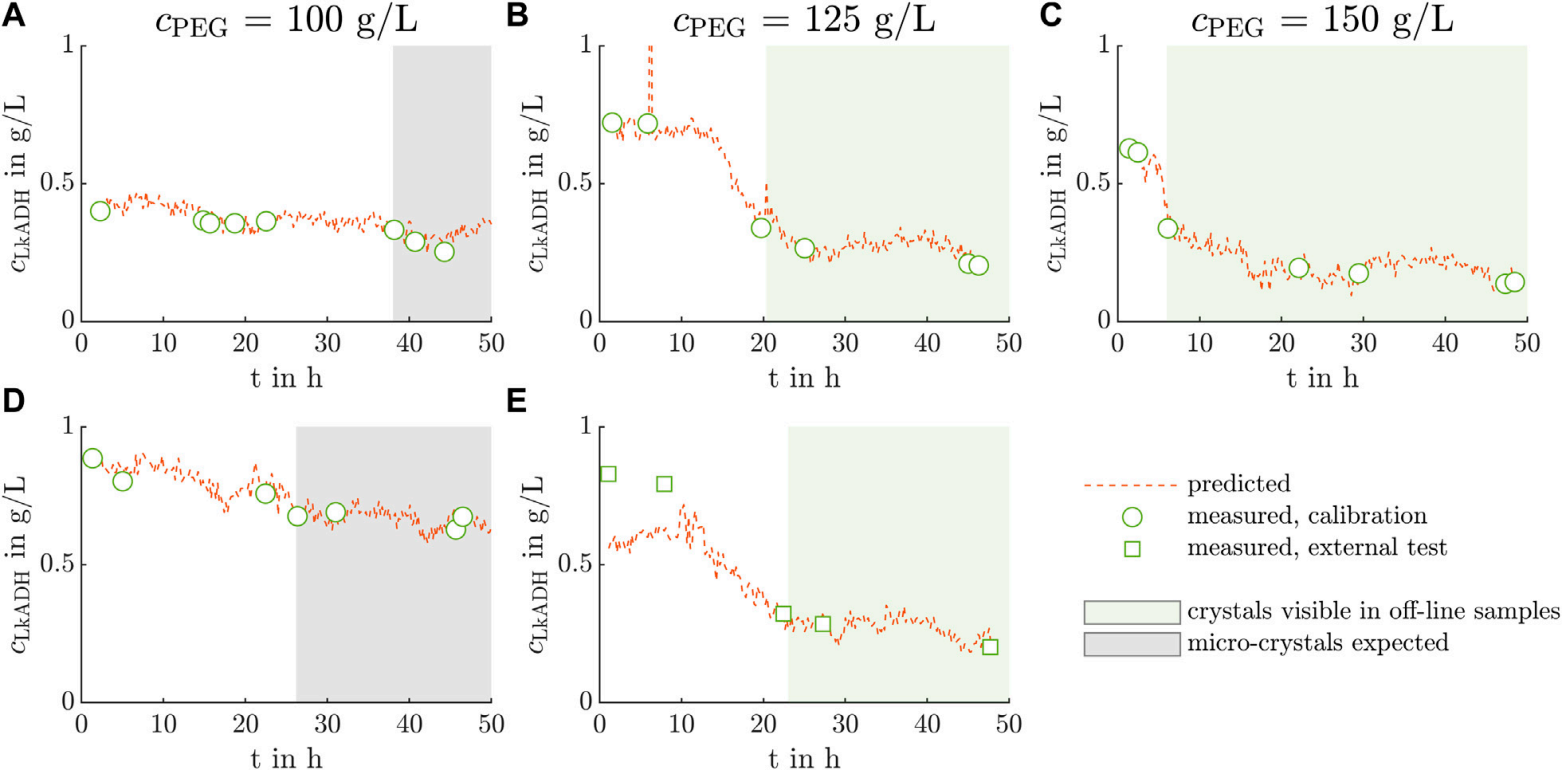
PLS model application to crystallization processes out of clarified lysate. The PLS model calculated with the manual data split predicts the *Lk*ADH concentration on the basis of the in-line Raman spectra in orange for the five conducted experiments **(A–E)**. Off-line *Lk*ADH calibration and validation concentrations are calculated from the IMAC analysis and are depicted by green circles and squares, respectively. Analogous to Figure 2, the light green boxes **(B, C, E)** indicate the time range when crystals are expected in the crystallization vessel as crystals are detected in the microscopic images of the off-line samples. The off-line samples in the time range illustrated by the gray boxes **(A, D)** showed only micro-crystals which were difficult to distinguish from precipitate visually.

The PLS model with manual data split is discussed in more detail in this section to emphasize the importance of data splitting. The results of the PLS model calibrated with the KS data split can be found in the Supplementary Material S8. The model predicted and measured *Lk*ADH supernatant concentrations of the five experiments are visualized in Figures 7A–E. Analogous, the results of the PLS model with a KS data split can be found in the Supplementary Material S8.

The white circles, gray squares, and orange lines represent the calibration, and validation concentrations measured off-line, as well as the in-line, model-predicted concentrations derived from the Raman spectra, respectively. The concentrations *c*_LkADH_ are shown over the time *t* with the start time being the moment when the crystallizing solution was added to the crystallization vessel. The gray shaded box indicates the time when crystals were assumed in the crystallization vessel as micro-crystals or crystals of a larger size were visible in the microscopic images of the off-line samples. The predicted data demonstrates fluctuations and outliers of up to 1.2 g L^−1^ in Figure 7B after 6 h. In Figures 7B,C,E, the predicted *Lk*ADH supernatant concentration decreases notably to 0.23 g L^−1^, 0.18 g L^−1^, and 0.24 g L^−1^ after 13 h, 5 h, and 10 h, respectively. After that, the concentration stays on the mentioned level fluctuating by 0.05 g L^−1^ Exp1 and Exp4 do not show a steep decrease in supernatant concentration, but a gradual decrease by 33% and 28% till the end of the experiment, respectively. Assessing the assumed crystal yield of the experiments Exp2, Exp3, Exp5 and the experiments Exp1, Exp4, the crystal yields were lower for crystallization conditions with decreasing *c*_PEG_ (see Table 1).

Comparing the model-predicted concentration values in Figures 7B,C,E, a *Lk*ADH concentration drop from the initial to the equilibrium concentration is clearly visible. In Exp2 and Exp5 with similar absorption values at 280 nm and PEG concentrations, crystallization required 12 h and 10.5 h, respectively, until the *Lk*ADH concentration decreased (see Figures 7B,E). The concentration decrease of Exp3, carried out at the highest PEG concentration of 150 g L^−1^, is visible after 4.5 h. The light green area indicates the off-line samples, in which crystals were visible in the microscopic images. This area starts after the concentration drop mentioned above, which indicates that protein crystallization caused the concentration drop. As the concentration drops in Exp1 and Exp4 are not pronounced, the time till the first detection of micro-crystals in the off-line samples was used for comparison. Micro-crystals were detected 11.7 h earlier in Exp4 than in Exp1 as the latter started with a lower 280 nm absorption value of the lysate and, thus, a lower, initial *Lk*ADH concentration influencing the supersaturation. When comparing the time till the first (micro)-crystals were visible, a trend towards shorter times with increasing PEG concentration becomes apparent.

## 4 Discussion

In this work, the implementation of an analytical bypass for particle-sensitive analytics and of an in-line Raman probe for batch protein crystallization process monitoring in real-time is discussed with the focus on their applicability and limitations. By means of the developed PAT and additional off-line analytics, the protein crystallization process itself can be assessed.

### 4.1 Analytical bypass and UV/Vis spectroscopy

To implement particle-sensitive analytics in a crystallization process, the analytical bypass consisted of a CFF-based set-up and enables monitoring the crystallization supernatant free of crystal particles. Inspecting the Supplementary Material S2, the recorded TMP and permeate flow rate showed irregularities and spikes which could be solved by stopping the CFF pump and cleaning the tubes manually. It is assumed that crystals or precipitated impurities blocked the membrane leading to a varying TMP levels in the experiments from day to day. Constant permeate flow rates indicate that the bypass and the sensor were filled with material representative of the liquid phase in the crystallization vessel. The comparison of Exp2 and Exp5 provides insight into the effects of the bypass on the crystallization process as the bypass was the only difference. Crystal breakage due to the CFF-based set-up can be excluded as the number, width, and height of the crystals were not reduced (see Figure 2, and Supplementary Material S3, S4). Other process characteristics, e.g., yield and purity, were not influenced as both experiments demonstrated comparable yields and a high HCP reduction factor (see Table 1).

Different levels of the initial UV/Vis absorption slope at 280 nm are visible in Figure 3A for Exp2, Exp3, and Exp4. A decreasing trend of the absorption slope is noticeable from day to day, but it does not directly correlate with the decreasing *Lk*ADH concentration in the supernatant obtained from the IMAC analysis of the off-line samples (data not shown). Note that impurities, e.g., nucleic acids or HCPs, were present (see Figure 3B and *c*_HCP, initial_ in Table 1) which absorb at 280 nm (Goldfarb et al., 1951; Gallagher, 2011) and aggravate direct absorption measurements at the selected wavelength. In Figure 3B Exp1 demonstrates an increased $\frac{A_{260nm}}{A_{280nm}}$ ratio of 1.6, which indicates a higher content of nucleic acids (Wilfinger et al., 1997) compared to the other experiments with a $\frac{A_{260nm}}{A_{280nm}}$ ratio around 1.0. The increased lysis cycle number may be the reason for this observation as more nucleic acids were released during a longer lysis duration and at higher energy input. The slight increase of the $\frac{A_{260nm}}{A_{280nm}}$ ratios from the first to the second day may be caused by crystallized protein which leads to a higher impurity proportion in the liquid phase. The decreasing $\frac{A_{260nm}}{A_{280nm}}$ ratio on the third day can be an effect of the insufficient permeate flow in the bypass. In our research, the analytical bypass provided information about the qualitative purity of the clarified lysate as changes in the starting material caused by the impurity composition could be detected.

In the past, Smejkal (2013)(Chapter 4.2) used a similar CFF-based set-up for automatic sampling during a crystallization process. However, this approach required sample dilution when the UV/Vis absorption value exceeded the detector saturation. The VP technology circumvents the additional dilution step and allows for automated UV/Vis analysis in real-time when the analytical bypass is switched on. Tian et al. (2023) described a different approach to implement UV/Vis spectroscopy as monitoring PAT for a pure lysozyme crystallization process using an ATR probe directly placed in the crystal slurry. However, ATR technology is limited to applications with strongly absorbing or highly concentrated solutions (Schlemmer and Katzer, 1987) and cannot adjust to concentration changes contrary to the VP technology. Furthermore, the real-time concentration could not be determined using the ATR UV/Vis absorption at 280 nm during the crystallization process because particle scattering obstructed the measurement (Van Eerdenbrugh et al., 2011) as soon as small crystals were formed (Tian et al., 2023). These challenges can be resolved by our approach separating the liquid phase from the crystals to implement UV/Vis spectroscopy in a protein crystallization process.

Taking into account the gained information about the supernatant composition by the $\frac{A_{260nm}}{A_{280nm}}$ ratio from the VP UV/Vis spectroscopy in the analytical bypass, the outliers of the bypass-related analytics, and the blockage of the tubing, the new insights by the UV/Vis spectroscopy did not justify the increased complexity of the bypass in the set-up for this project. The variation in the UV/Vis spectrum could not be correlated with process parameters, e.g., target molecule concentration in the supernatant, as the data usability was lowered by interfering impurities in the UV/Vis spectrum, lacking overnight data, and difficulties during the start of the bypass. However, the implementation of other particle-sensitive analytics should be possible, e.g., fluorescence or nuclear magnetic resonance (NMR) spectroscopy, if applicable in the specific crystallization process.

### 4.2 Raman spectroscopy and chemometrics

To characterize and potentially monitor crystallization, a Raman spectroscopy probe was immersed directly into the stirred crystallization vessel. The probe was in direct contact with the crystal suspension and may show variations in the spectrum over the process time as the liquid phase composition changes.

Spectral differences are visible between 450 and 1,500 cm^−1^ and between 2,800 and 3,000 cm^−1^. The latter is attributed to C-H stretching (Bocklitz et al., 2011). The former is described as the fingerprint region of proteins (Bocklitz et al., 2011). Comparing the spectra to Raman spectra of air, protein buffer, and crystallization buffer, preprocessed Raman peaks could be traced back to different compounds. The crystallization buffer spectrum shows distinct peaks near 850, 1065, 1140, 1250, 1,286, and 1,475 cm^−1^ (see Supplementary Material S5B). As PEG contributes strongly to the Raman spectrum compared to the protein, the spectral analysis is hampered with respect to the desired process characteristics, i.e., crystal yield and target molecule concentration in the supernatant. Minor differences are visible between the spectra of the crystallization buffer and during the experiment near 970–1,030 cm^−1^ and between 1,170 and 1,230 cm^−1^. This may be caused by the amino acid contribution of Phenyalanine (Phe) (1000, 1030, and 1,205 cm^−1^ Tuma, 2005; Huang et al., 2006) and Tyrosine (Tyr) (1,174, 1,205 cm^−1^ Tuma, 2005). The wavenumbers 757, 853 cm^−1^, and 1,225–1,525 cm^−1^ are associated with Tryptophan (Try), Tyr, and the amide III bands, respectively (Huang et al., 2006). The amide III bands are further associated with the wavenumber range 1,220–1,400 cm^−1^ (Kuhar et al., 2021). In low frequency ranges, the amino acids Tyr and Phe affect the Raman spectrum near the wavenumbers 336, 417 cm^−1^, and 476 cm^−1^(Overman and Thomas, 1999). To emphasize minor, spectral differences, suitable preprocessing, e.g., spectral derivative calculation, are advised.

The chemometric analysis of the preprocessed Raman spectra with PCA showed that the experiments followed a linear trend (see Figure 5). Note that Exp3 showed a faster transition from the lower right to the upper left corner as indicated by the center of observations (see Figure 5C). It may be linked to crystallization accompanied by a decreasing *Lk*ADH concentration. The PCA of the UV/Vis spectra could only cluster the experiments according to the experiment conditions of lysis cycle number and varying initial absorption of the clarified lysate at 280 nm, but did not show a trend within each experiment (data not shown). Five peaks at 493 cm^−1^, 708 cm^−1^, 1,410 cm^−1^, 2,909 cm^−1^, and 2,970 cm^−1^ occurred during Exp2 and Exp5 (see Figure 4B) and could not be traced back to protein crystals. Looking at the PC1 and PC2 of the PCA over the whole spectral range, the supposedly defective observations are captured in clusters which are clearly separated from the crystallization-associated, linear trends (see Figures 5B,E). As these peaks did not correlate with the protein buffer or crystallizing buffer, the *Lk*ADH concentration in the supernatant, or the crystal yield and appeared or disappeared spontaneously, it is assumed that precipitated impurities may have aggregated and accumulated near or detached from the spectroscopy probe arbitrarily due to the agitation in the stirred vessel. The authors exclude scattering crystals as the potential cause as the spectral interference occurred and disappeared randomly. Crystals were detected in experiments without the peak irregularities (see Figure 2C). Furthermore, the drop of *Lk*ADH concentration in the supernatant, indicating the start of the crystallization process, did not coincide with the appearance of the spectral interferences.

Based on these findings, the Raman spectra and the presented preprocessing procedure were used for PAT model development. The wavenumber ranges above (see Figure 4C) are selected for the PLS model development as they are assumed to correlate with product characteristics, *Lk*ADH concentration for instance.

The KS algorithm-based and manual data split with Exp5 were investigated and compared to evaluate the extrapolation capability of the calculated PLS models. The metrics for chemometric models demonstrate a high model validity in both model cases (see Figure 6). The values *R* ^2^ and *Q* ^2^ are near 1, implying a good transferability to the external spectral data set. *RMSECV* and *RMSEP* are desired to be low. In this case, the PLS model with KS data split demonstrates higher *R* ^2^, *Q* ^2^ and lower *RMSEP* values - both suggesting that the PLS model with KS data split is superior. The KS data split method depends on the specified number of validation samples and selects the validation samples based on a uniform distribution of the data split using a distance metric. The assumption of the data being split into highly similar data subsets may not hold in all situations, limiting its applicability to certain types of data. The spectra in the second cluster of the PCA were not included in the validation set by the KS algorithm, which improved the model evaluation metrics. Data points of only Exp1, and one data point of Exp2 and Exp4 each are selected to represent the validation data set (see Supplementary Material S8). The calibration of the model based on data of each experiment may cause the model to potentially incorporate variations of the experiments and batch-to-batch variations. Batch-to-batch variations are caused during the *Lk*ADH production in *E. coli*, by variations during lysis and clarification, and, in our case, the different initial crystallization conditions (see Table 1). To find out whether the model can be applied for extrapolation of a new experiment, the authors decided to proceed with the model calibrated with the manual data split using Exp5 for validation. In this way, the PLS model prediction performance could be evaluated on data possibly prone to experimental variations.

The model calibrated and validated with the manual data split underestimated the *Lk*ADH concentration in Exp5 at higher concentrations above 0.7 g L^−1^ (see Figure 7E). Within the first 10 h, crystallization was not visible in the microscopic images of the off-line samples. The Raman spectrum may be influenced by other processes occurring in the crystallization vessel, which leads to an underestimated concentration prediction in the first discussed time slot. The spectra of samples with high *Lk*ADH concentration in Exp4 were not representative of spectra in Exp5. As the *Lk*ADH concentration was at a comparable level, other species present in the supernatant may interfere. The crystallization solution contains PEG which is known to induce aggregation or precipitation. Aggregation processes of impurities, namely, nucleic acids or HCPs, in the examined time slot may affect the Raman spectrum and lead to the visible discrepancies.

At lower concentrations, the model performed well, even though the spectra of Exp5 varied strongly (see the second cluster in Figure 5E compared to A-D). The selected preprocessing parameters and wavenumber ranges were able to cope with the disturbance in lower concentration ranges. However, off-line samples and their analysis cannot be left out entirely, but the sample number may be reduced by combining Raman spectroscopy with PLS modeling for protein crystallization PAT. More experiments with varied crystallization conditions, more samples analyzed, and different cultivation batches can increase the variety of spectra and reduce the effect of outliers, which is beneficial for model calibration. This may lead to a better chemometric model producing reliable predictions over the whole concentration range.

### 4.3 Assessment of the crystallization process using multiple PAT tools

The presented analytical set-up as a whole provides the possibility to examine the conducted experiments regarding the type and amount of impurities, target molecule, and different initial absorption values at 280 nm. The shorter time of *Lk*ADH decrease in the supernatant with increasing PEG concentration marks the start of protein crystallization (see Figure 7 from left to right) as the PEG concentration influences the phase behavior and the supersaturation (Galkin and Vekilov, 2001). Baumgartner et al. (2015) examined the effect of two PEG additives on different proteins and observed an increasing depletion attraction effect with increasing polymer concentration (Vivarès et al., 2002). The enzyme *Lactobacillus brevis* alcohol dehydrogenase (*Lb*ADH) which is a homologous protein to *Lk*ADH of this project showed an increasing tendency to form crystals with increasing PEG concentration and was studied in detail in Nowotny et al. (2019). Furthermore, the PEG concentration influences the crystal geometry and leads to larger crystal sizes (see Supplementary Material S4) at a lower supersaturation level (McPherson and Cudney, 2014; Klijn and Hubbuch, 2018) in a crystallization buffer with 125 g L^−1^ PEG in Exp2 and Exp5. These experiments led to the highest crystal counts per well in Figure 2. This contradicts the fact that higher supersaturation levels result in a larger amount of smaller crystals, but can be explained by the fact that smaller crystals are difficult to detect automatically by the ML-based tool due to the low ratio of the crystal size to the camera resolution.

The presence of impurities is reflected by the nucleic acid and HCP content in Figure 3B and in Table 1, respectively. The HCP reduction of a similar magnitude was achieved for crystal redissolution of a homologous enzyme (Nowotny et al., 2019). Even though the $\frac{A_{260nm}}{A_{280nm}}$ ratio and HCP quantification are based on on-line and off-line analytics, they provide valuable process knowledge and help to understand crystallization processes in complex, heterogeneous solutions.

At a higher absorption *A*_280nm_ of the clarified lysate in Exp4, a high *Lk*ADH concentration was achieved in the beginning of the crystallization process (see Figure 7D). Compared to Exp1 at the same PEG concentration, protein crystals could be detected earlier, but the supernatant concentration of *Lk*ADH did not drop to the same value. It is assumed that the equilibrium and the maximum crystal yield were not reached within the duration of the experiment. These findings contradict the results of Walla et al. (2021) who observed that *Lk*ADH WT reached the equilibrium within 48 h for the screened PEG concentration. Note that the analytical frame differed in the mentioned project as the total protein concentration was determined. The crystal yields of the experiments performed at 125 or 150 g L^−1^ PEG were lower than the yields achieved in Walla et al. (2021). In this work, the crystal yield and crystallization process time were derived from the *Lk*ADH concentration with IMAC, which makes a direct comparison difficult. Furthermore, variations during the cultivation, lysis, the lysate clarification procedure, or crystallization vessel (see Supplementary Table S1) may change the product or impurity profiles and lead to a lower, initial *Lk*ADH concentration when the *A*_280nm_ is adjusted to the same value. In future crystallization experiments, the presented monitoring set-up may be applied to other crystallization processes in biological, complex solutions resulting in new knowledge on crystal yield and crystallization process time.

## 5 Conclusion

This research project aimed to examine and monitor stirred *L. kefir* alcohol dehydrogenase (*Lk*ADH) enzyme crystallization out of clarified *E. coli* (*E. coli*) lysate on a 300 mL scale to increase process understanding of a multi-phase process. The implemented analytics included an in-line Raman spectroscopy probe, on-line cross-flow filtration (CFF) bypass for the liquid phase analysis in a variable path length (VP) flow cell for ultraviolet visible light (UV/Vis) spectroscopy, high-performance liquid chromatography (HPLC) immobilized metal ion affinity chromatography (IMAC), enzyme-linked immunosorbent assay (ELISA), and microscopic analysis of off-line samples.

Chemometric analysis of the preprocessed Raman spectra could identify similar process trends in the spectra of the experiments with principal component analysis (PCA) and could monitor the *Lk*ADH concentration in clarified lysate with a partial least squares (PLS) regression model built on selected wavenumber regions containing product-relevant information. The presented analytical set-up provided a comprehensive overview of the conducted batch experiments, which is in agreement with theoretical considerations of protein crystallization.

Despite the complexity of the clarified lysate, a suspension containing scattering crystals, impurities in the supernatant, and precipitate, spectroscopy could be used to monitor the target molecule concentration in the liquid phase during a multi-phase process. The analytical bypass facilitated the implementation of particle-sensitive analytics, i.e., VP UV/Vis spectroscopy, which indicated changes in the contaminant profile with the absorption ratio at two specific wavelengths typical for proteins and nucleic acids. The off-line analysis of microscopic images allowed for an objective evaluation of crystal presence or crystal breakage. In our case, the crystal number and geometry did not vary when a CFF bypass was installed meaning that crystal breakage was not observed at the chosen CFF process parameters. In terms of model limitations, batch-to-batch variations and the heterogeneous components in the clarified lysate aggravated the direct model transfer to new experiments without additional validation samples.

The process analytical technology (PAT) set-up with in-line Raman spectroscopy can be applied to other processes based on phase behavior, e.g., precipitation or flocculation, if the molecule of interest contributes to the recorded spectrum sufficiently. A good calibration procedure and carefully considered data splitting for the model development help to unravel the underlying spectral nuances associated with the desired product characteristics. The increased process understanding and possibility to monitor phase behavior based processes can help the operator optimize the process, e.g., regarding crystal yield. When protein solutions of high purity need to be crystallized, the installation of an on-line bypass with VP UV/Vis measurements can be especially useful to directly determine the supernatant concentration. Monitoring protein crystallization processes is essential for process control and process adaptations as biotechnological processes are often subject to batch-to-batch variability.

## Data Availability

The raw data supporting the conclusion of this article will be made available by the authors, without undue reservation.
