# Supplementary material for: Spectroscopic insights into multi-phase protein crystallization in complex lysate using Raman spectroscopy and a particle-free bypass
Source: Front Bioeng Biotechnol. 2024 May 15;12:1397465. doi: 10.3389/fbioe.2024.1397465 (PMC11133712; doi:10.3389/fbioe.2024.1397465)
Supplement: Supplementary file 1 [file DataSheet1.PDF]

Supplementary Material

1 SUPPLEMENTARY TABLES

1.1 Variations of *Lk*ADH production and preparation compared to Walla et al. (2021)

Table S1. Differences between the protein production, preparation, and crystallization experiment: The experimental and equipment variations between the crystallization experiments of this research work and in Walla et al. (2021) are listed.

process step	variations	material & methods sections 2.1 and 2.2	Walla et al. (2021)
cultivation	mode<br>vessel	fed-batch<br>1.5 L parallel fermenter	batch<br>0.5 L shake flasks
cell lysis	device<br>microtip<br><br><br><br>amplitude<br>pulse<br>pulse time<br>cycle number	Sonifier SFX550<br>tapered Microtip 101-148-062<br>(Branson Ultrasonic Corporation)<br><br>70 %<br>10 s<br>40 s<br>2 or 3	Sonoplus HD 2070<br>Microtip MS 72<br>(BANDELIN electronic, GmbH & Co. KG)<br><br>90 %<br>0.5 s<br>90 s<br>3
dialysis	membrane<br><br><br>MWCO<br>ID	SnakeSkin™<br>(Thermo Fisher Scientific, Inc.)<br><br>3.5 kDa<br>22 mm	Membra-Cel(TM) Cellu.<br>(Carl Roth GmbH + Co. KG)<br><br>14 kDa<br>34 mm
crystallization	scale<br>stirrer speed<br>stirrer geometry	300 mL<br>80 rpm<br>anchor style paddles	5 mL<br>150 rpm<br>pitched-blade impellers

2 SUPPLEMENTARY FIGURES

2.1 IMAC analysis

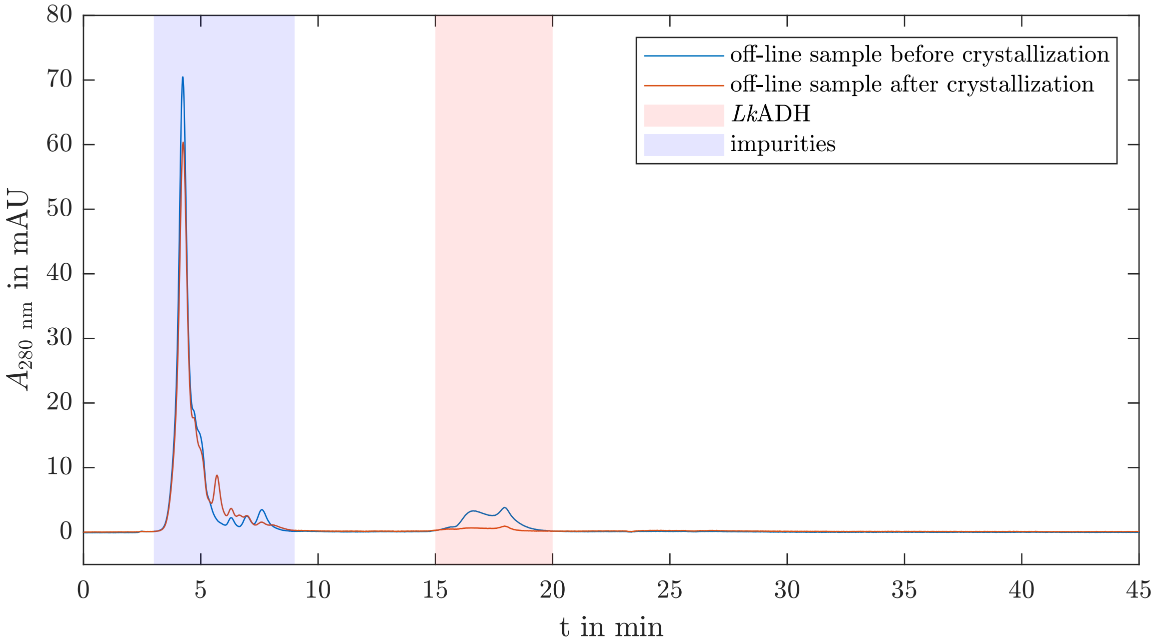

Figure S1. Exemplary separation with high-performance liquid chromatography (HPLC) immobilized metal ion affinity chromatography (IMAC). The absorption at $280\,\mathrm{nm}$ of the IMAC analysis is shown over time. The blue shaded area indicates the impurities in the flow-through whereas the red shaded area is the *Lactobacillus kefir* alcohol dehydrogenase (*Lk*ADH) peak which elutes with increased imidazole concentration. Exemplarily, two off-line samples at the beginning and end of the experiment are depicted in blue and orange.

2.2 CFF characterization

The transmembrane pressure (TMP) over the cross-flow filtration (CFF) membrane in Figure S2, as well as the flow rate of the permeate stream, can be used to assess the reliability of the on-line sensor in the analytical bypass. The sensor can only measure reliably if the solution in the bypass represents the current particle-free vessel contents. The TMP for all experiments was mainly below $0.15\,\mathrm{bar}$ and remained constant for several hours on each day. Outliers are visible in all experiments which occurred when the bypass was blocked requiring manual blockage removal. As the bypass was only operated during the day, data is missing in the nighttime. Exp2, Exp3, and Exp4 do not demonstrate stable TMP values on the third day and, consequently, the bypass was switched off. The start and stop time varied as the experiments started at different time points during the day.

The flow rates in the permeate stream in all experiments drop from a value between $3\,\mathrm{mL\,min^{-1}}$ to $6\,\mathrm{mL\,min^{-1}}$ to a value of $0.1\,\mathrm{mL\,min^{-1}}$ to $1.1\,\mathrm{mL\,min^{-1}}$ within the first four hours of each experiment. On the second and third day, the flow rate of Exp1 and Exp3 remain on a constant level between $0.01\,\mathrm{mL\,min^{-1}}$ to $1.1\,\mathrm{mL\,min^{-1}}$. The flow rate values for Exp2 and for Exp4 fluctuate between $-0.5\,\mathrm{mL\,min^{-1}}$ to $0.5\,\mathrm{mL\,min^{-1}}$.

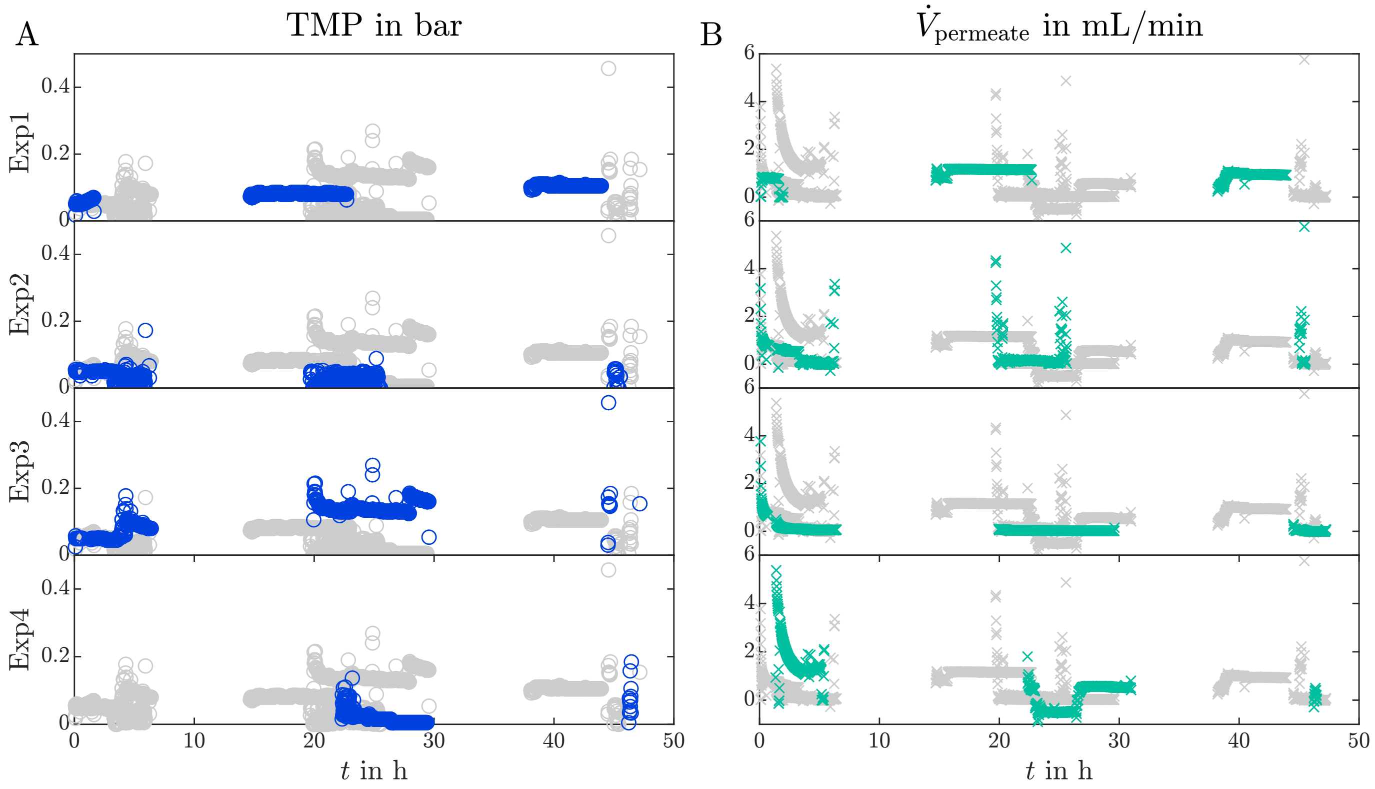

Figure S2. Characterization of the analytical by-pass. The analytical bypass can be characterized by the measurement of the TMP (A) or permeate flow rate $\dot{V}_{\mathrm{permeate}}$ (B) over time for Exp1 - Exp4. They are visualized with blue circles and turquoise crosses. For clearer visualization, TMP and the $\dot{V}_{\mathrm{permeate}}$ are averaged with a moving mean over each minute and only one value per minute is shown as the data were recorded with a high frequency. The missing data are caused by the fact that the bypass was switched off overnight. Spikes in the recorded flow rate are artefacts from starting or turning off the analytical bypass.

2.3 Machine-learning-based image analysis

2.3.1 Interpretation of the machine-learning-based image analysis results

Information about the crystal geometry need to be interpreted carefully bearing in mind the actual number of counted crystals (see Figure 2). The determined crystal widths and heights when low numbers or no crystals were visually detected can be caused by the high noise level in the images when the model falsely detects crystals. Especially the results of the automated image analysis of the experiments conducted with polyethylene glycol (PEG) concentration of $10\,\%$ may be prone to false-positive crystal detections as microcrystals can be expected, but larger crystals were not visible by human eye. The authors interpreted the provided information about the geometry in the Figures S3 and S4 as an indication that the crystal geometry does not change over time when larger crystal counts were reached.

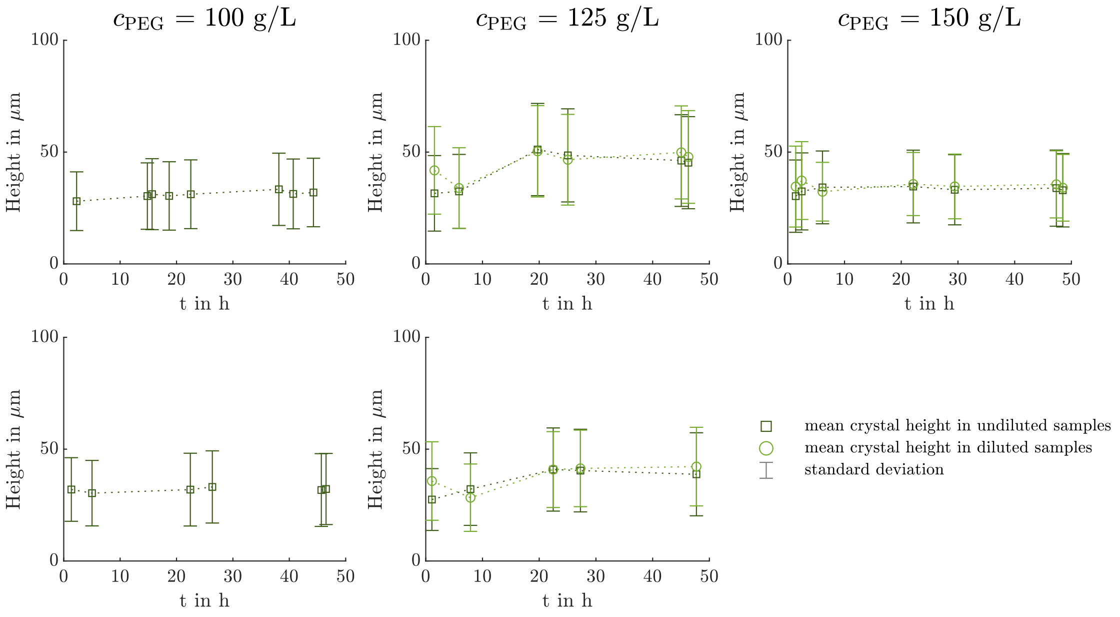

Figure S3. The automated machine learning (ML)-based image analysis (Bischoff et al., 2022) detected crystals, and determined the crystal height and width. These information can be used to characterize the crystal geometry throughout the experiments, i.e. crystal height and width. The **mean crystal height**, and the standard deviation in the undiluted and diluted off-line samples are depicted over time for five experiments in dark green squares and light green circles, respectively.

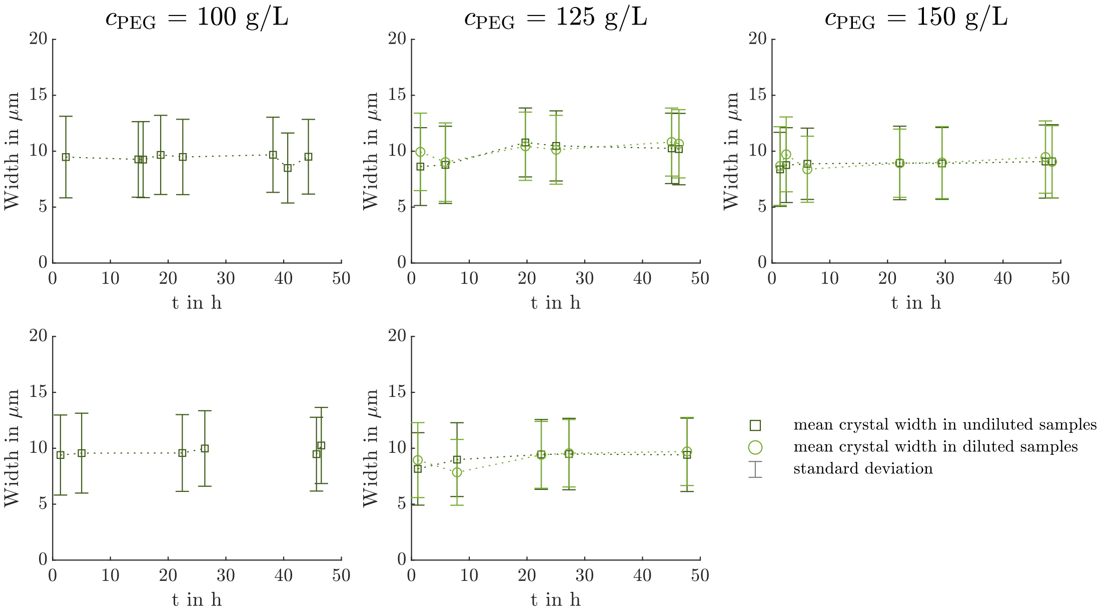

Figure S4. The automated ML-based image analysis (Bischoff et al., 2022) detected crystals, and determined the crystal height and width. These information can be used to characterize the crystal geometry throughout the experiments, i.e. crystal height and width. The **mean crystal width**, and the standard deviation in the undiluted and diluted off-line samples are depicted over time for five experiments in dark green squares and light green circles, respectively.

2.4 Background Raman spectrum of protein and crystallization buffer

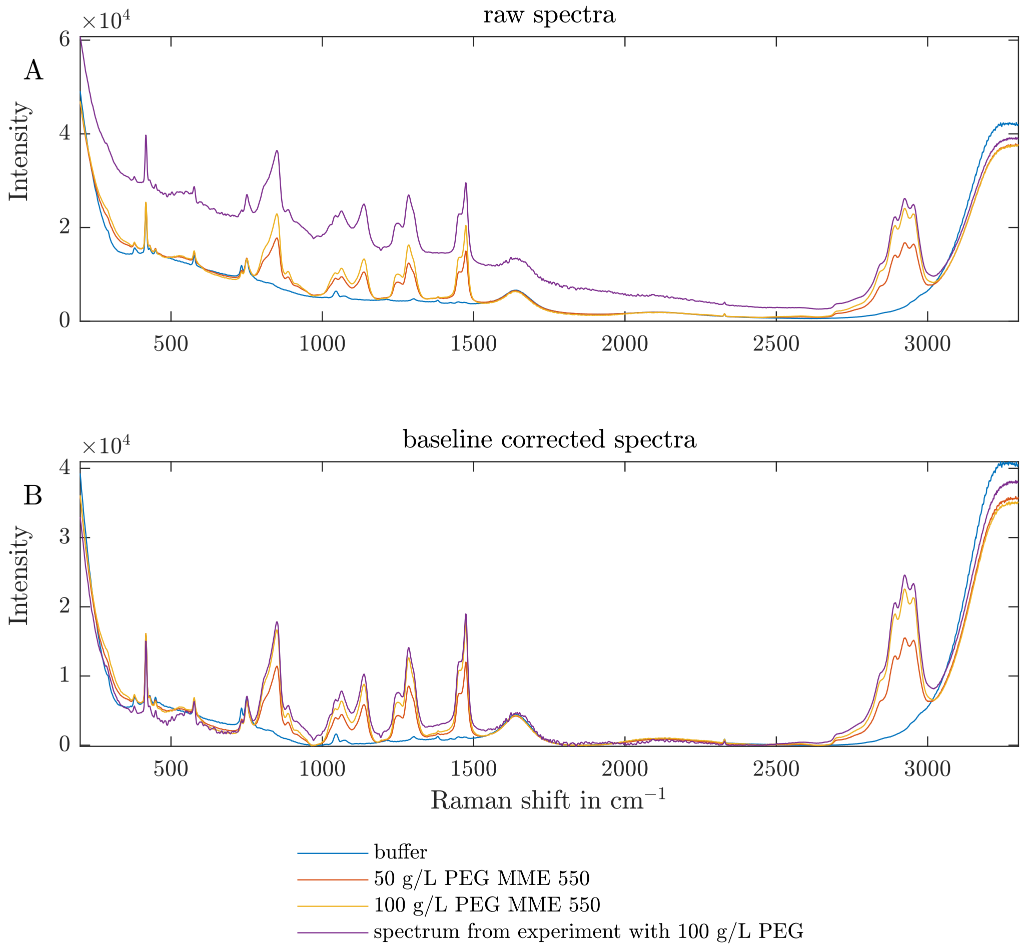

Figure S5. The Raman intensity of protein buffer, crystallization buffer with PEG at different concentrations, and one spectrum derived from an experiment are shown over the wavenumber shift in blue, orange, yellow and purple line color, respectively. The raw and preprocessed spectra after baseline-correction are visible in (A, B).

2.5 Zoom into the preprocessed spectra of Exp3

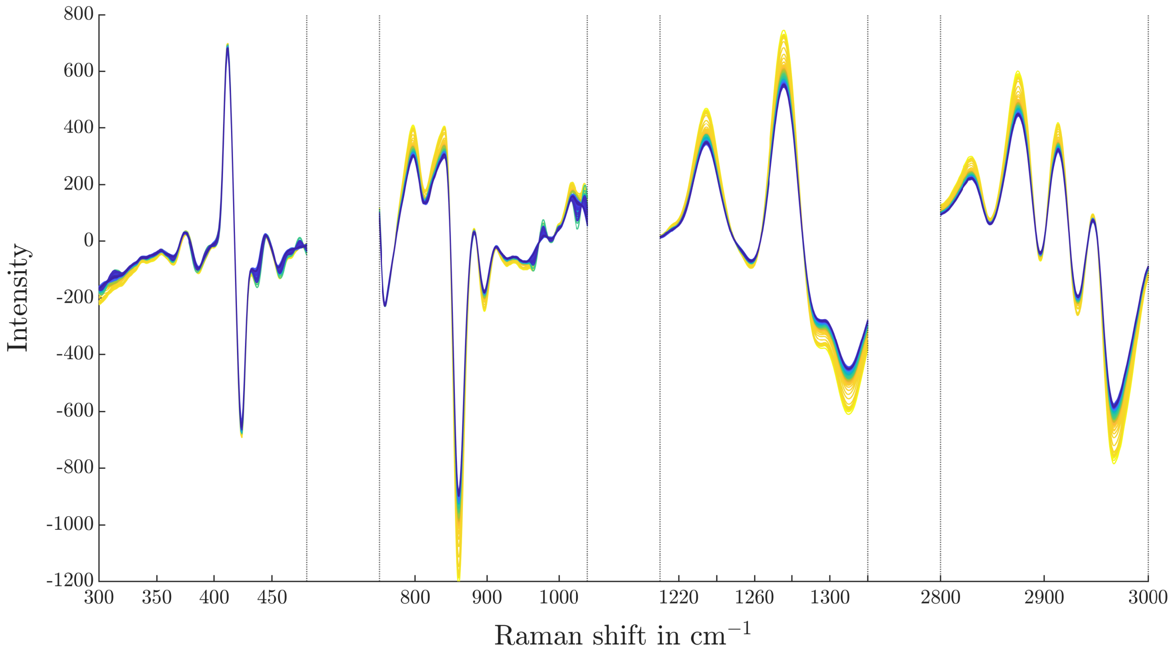

Figure S6. The preprocessed Raman spectra of Exp3 are illustrated over the selected wavenumber regions for the partial least squares (PLS) model with the manual data split. The time course of the experimental spectra is visualized from yellow to blue.

2.6 PCA loadings

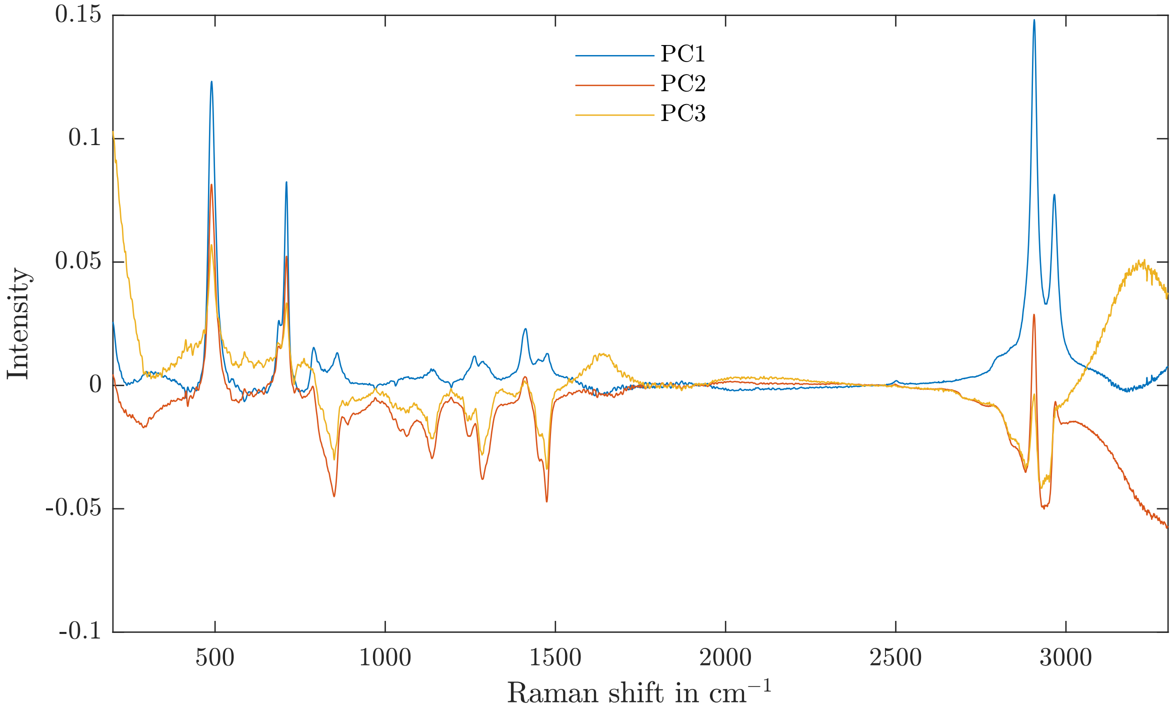

Figure S7. The principal components PC1, PC2, and PC3 of baseline-corrected Raman spectra of all experiments are illustrated over the recorded wavenumber range in blue, red and yellow, respectively.

2.7 PLS model with KS algorithm applied on crystallization process spectra

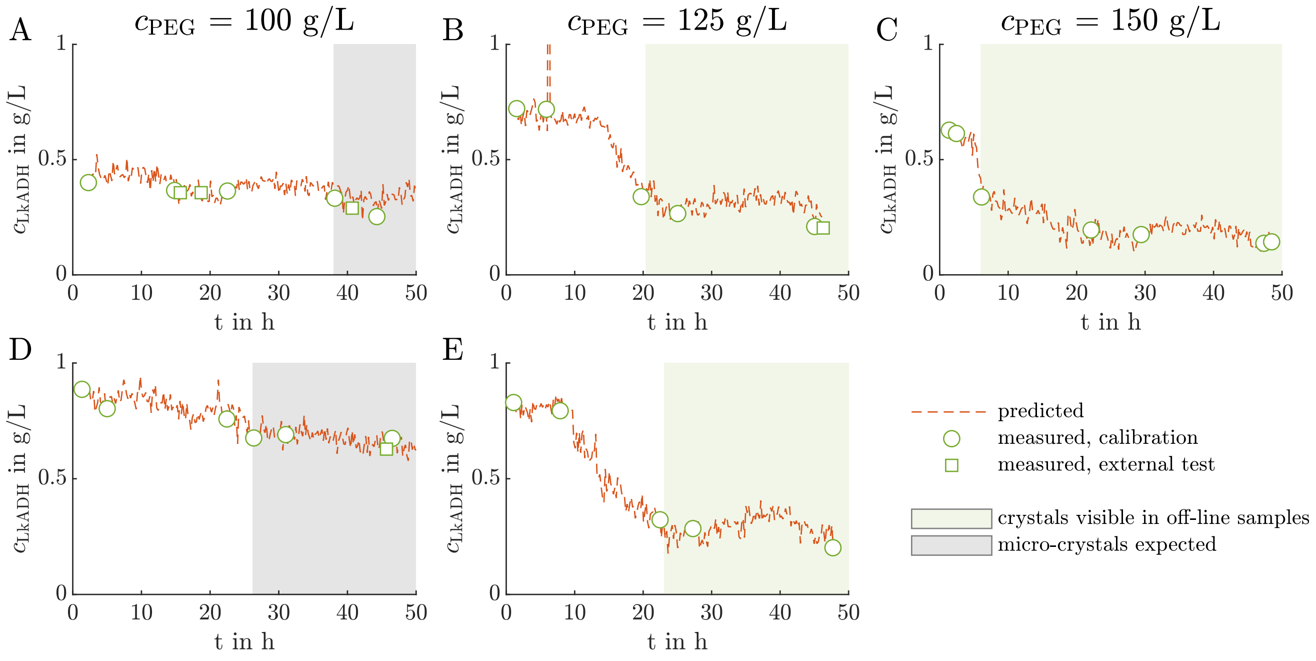

Figure S8. Application of PLS model on crystallization processes out of clarified lysate. The calculated PLS model with the Kennard-Stone (KS) validation data split predicts the *Lk*ADH concentration on the basis of the in-line recorded Raman spectra in orange for the five conducted experiments (A-E). Off-line *Lk*ADH calibration and validation concentrations are calculated from the IMAC analysis and are depicted with green circles and squares, respectively. The light green boxes indicate the time range when crystals were expected in the crystallization vessel as crystals were detected in the microscopic images in the off-line samples. The light gray boxes indicate time ranges in the Exp1 and Exp4 experiment when only micro-crystals were visible in the microscopic images which are difficult to distinguish from precipitate.

REFERENCES

Bischoff, D., Walla, B., and Weuster-Botz, D. (2022). Machine learning-based protein crystal detection for monitoring of crystallization processes enabled with large-scale synthetic data sets of photorealistic images. *Analytical and Bioanalytical Chemistry* 414, 6379–6391. doi:10.1007/s00216-022-04101-8

Walla, B., Bischoff, D., Janowski, R., Von Den Eichen, N., Niessing, D., and Weuster-Botz, D. (2021). Transfer of a rational crystal contact engineering strategy between diverse alcohol dehydrogenases. *Crystals* 11, 8–12. doi:10.3390/cryst11080975
